# Tin-selenide as a futuristic material: properties and applications

**DOI:** 10.1039/d0ra09807h

**Published:** 2021-02-10

**Authors:** Manoj Kumar, Sanju Rani, Yogesh Singh, Kuldeep Singh Gour, Vidya Nand Singh

**Affiliations:** Academy of Scientific and Innovative Research (AcSIR), CSIR- Human Resource Development Centre, (CSIR-HRDC) Campus Ghaziabad Uttar Pradesh 201002 India singhvn@nplindia.org; Indian Reference Materials (BND) Division, National Physical Laboratory, Council of Scientific and Industrial Research (CSIR) Dr K. S. Krishnan Road New Delhi 110012 India; Optoelectronics Convergence Research Center, Chonnam National University Gwangju 61186 Republic of Korea

## Abstract

SnSe/SnSe_2_ is a promising versatile material with applications in various fields like solar cells, photodetectors, memory devices, lithium and sodium-ion batteries, gas sensing, photocatalysis, supercapacitors, topological insulators, resistive switching devices due to its optimal band gap. In this review, all possible applications of SnSe/SnSe_2_ have been summarized. Some of the basic properties, as well as synthesis techniques have also been outlined. This review will help the researcher to understand the properties and possible applications of tin selenide-based materials. Thus, this will help in advancing the field of tin selenide-based materials for next generation technology.

## Abstract 2

SnSe/SnSe_2_ has diverse applications like solar cells, photodetectors, memory devices, Li and Na-ion batteries, gas sensors, photocatalysis, supercapacitors, topological insulators, resistive switching devices due to its optimal band gap.

## Graphical abstract

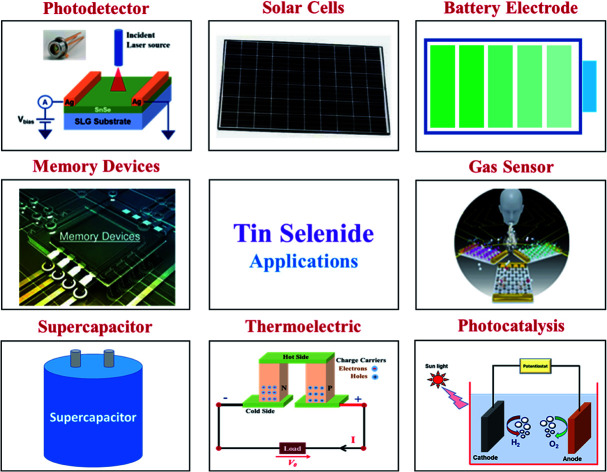

## 1. Introduction

The fast developing area of applied material science demands materials to be cheap, non-toxic, environment-friendly, easy to synthesize, and well competitive in performing particular applications. Nowadays, materials with versatility have gained massive attention due to their applicability in almost all fields.^1,2^ Various materials have been explored and showed promising versatile applications, *e.g.*, graphene (Gr),^1^ TiO_2_,^3^ ZnO,^4^ Cu_2_SnS_3_ (CTS),^2^*etc.* The multifunctional applicability of these materials paves the foundation for interdisciplinary research. Chalcogenide-based materials have also shown such potential and can be seen as the future hope to meet a similar requirement. Among chalcogenides, tin selenide has demonstrated great potential in the applied material science. Tin selenide exists in two phases, *i.e.*, SnSe and SnSe_2_. Some researchers have observed another phase, Sn_2_Se_3_,^5,6^ but this phase is the superposition of SnSe and SnSe_2_.^7^ Tin selenide has demonstrated versatility in thermoelectric,^8^ photodetector,^9^ solar cells,^10^ photocatalytic,^11^ phase change memory,^12^ gas sensing,^13^ anode material for battery,^14^ supercapacitor,^15^ and topological insulator (TI).^16^ These applications strongly depend upon the properties of SnSe (optical, electrical and microstructural, *etc.*). Apart from material properties, material synthesis/deposition methods also play an essential role in obtaining high-quality materials.^17–21^ Excellent review article exists on thermoelectric materials consisting of fundamental properties to the thermoelectric device's final design, growth, defects, working environment issues, and applications.^22^ Other reviews that focusses on SnSe describes all the aspects mentioned above (like growth, defects, configuration, *etc.*).^23^ The aim of this review is to summarize the ongoing progress on SnSe, SnSe_2_ synthesis methods, materials properties, and its possible application in various fields. However, some studies focus on pristine tin-selenide phase and its application^8,24^ Another good reviews^8,25,26^ that concentrates on SnSe describing all aspects mentioned above (like growth, defects, design, *etc.*), provides all-round knowledge to researchers. This review gives insights into the phases, structures, synthesis methods, progress in the tin-selenide, and tin diselenide for various applications. Hence, this article aims to review the tin selenide devices, present status, recent progress in the growth process, related-issues, possible solutions, and their possibility of developing next-generation technology.

## 2. Material properties of tin selenide

**Fig. 1:**
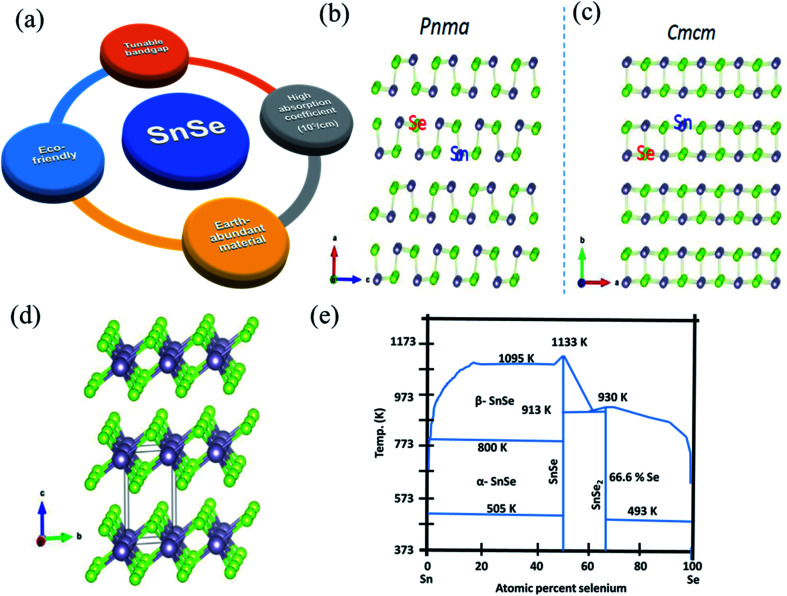
(a) Salient feature of tin selenide materials. (b) Crystal structure of SnSe, (c) SnSe having *Pnma* to *Cmcm* phase transition. This figure has been adapted/reproduced from ref. 38 with permission from Elsevier, copyright 2018". (d) Crystal structure of SnSe_2_. This figure has been adapted/reproduced from ref. 33 with permission from ACS, copyright 2016". (e) Equilibrium phase diagram of Sn–Se system. This figure has been adapted/reproduced from ref. 39 with permission from Wiley, copyright 2020".

Tin selenide based SnSe/SnSe_2_ are binary compound semiconductor materials having p/n-type conductivity.^20,27^Fig. 1(a) shows a salient feature of tin selenide materials. The SnSe exists in two crystallographic phases, *i.e.*, orthorhombic (α-SnSe)^28^ and cubic (π-SnSe),^29^ and SnSe_2_^ 30^ exists in the hexagonal crystal structure. The π-SnSe material is mechanically stable and energetically comparable to α-SnSe and has already shown potential in the piezoelectric application.^29^ The orthorhombic crystal structure of SnSe showed second-order displacive type phase transition above 750 K, from α-SnSe (space group *Pnma*) to β-SnSe (space group-*Cmcm*) with (*a* = 11.49 Å, *b* = 4.44 Å, *c* = 4.135 Å) to the (*a* = 4.31 Å, *b* = 11.70 Å, *c* = 4.31 Å), respectively (Fig. 1(b)).^31,32^ The crystal structure of SnSe (violet, Sn atoms; green Se atoms) (Fig. 1(c)),^32^ and crystal structures of SnSe_2_ (green, Se atoms; violet, Sn atoms) is shown in Fig. 1(d).^33^ Both the tin-selenide phases show the indirect and direct band gaps. The direct band gap of α-SnSe shows a wide tunable band gap which varies from 0.98 eV (bulk) to 1.43 eV (monolayer).^34^ SnSe_2_ shows large variation in the band gap from 1.84 eV (bulk) to 2.04 eV (monolayer).^35^ This band gap tunability of tin selenide shows its immense application possibilities in optoelectronic device applications.^30,36^ Tin selenide exists in two stoichiometric phases, *i.e.*, SnSe and SnSe_2_, as shown by the equilibrium phase diagram (Fig. 1(e)).^7^ Various research groups have reported another phase, Sn_2_Se_3_,^37^ but this phase is the superposition of the SnSe and SnSe_2_^ 7^ as confirmed by Nuclear Magnetic Resonance (NMR) spectroscopy.

Defects present in the material also influences the properties of materials like electronic, magnetic, and optical properties. SnSe is generally a p-type semiconductor. Density functional theory (DFT) calculation is performed to study the defects during SnSe crystal growth. The Sn vacancy is present as a native defect, which causes the p-type conduction in the SnSe under Se or Sn rich conditions, as confirmed by Scanning Tunneling Microscopy (STM) studies.^40,41^ SnSe_2_ shows n-type conduction. DFT calculation shows that the vacancy of selenium and interstitial tin led to this n-type conduction in SnSe_2_.^41^ The optoelectronic and physical properties of SnSe/SnSe_2_ materials depend on the growth conditions, size, morphology, phase purity, growth techniques, *etc.* Therefore, selection of synthesis/deposition method impacts material as well as its proposed applications.

## 3. Synthesis of SnSe and SnSe_2_

The material's properties also depend on the growth conditions,^42^ size, and morphology of the material,^43^ phase purity,^43^ defects,^44^*etc.* Various physical and chemical methods have been established to fabricate SnSe/SnSe_2_ materials on different substrates (glass, flexible metal foil, polymer, *etc.*) for multiple applications. The tin selenide based semiconductor materials have been synthesized using atomic layer deposition (ALD),^17^ sputtering,^45^ thermal evaporation,^46^ hydrothermal,^47^ spray pyrolysis,^48^ chemical vapor deposition (CVD),^49^*etc.*

**Fig. 2:**
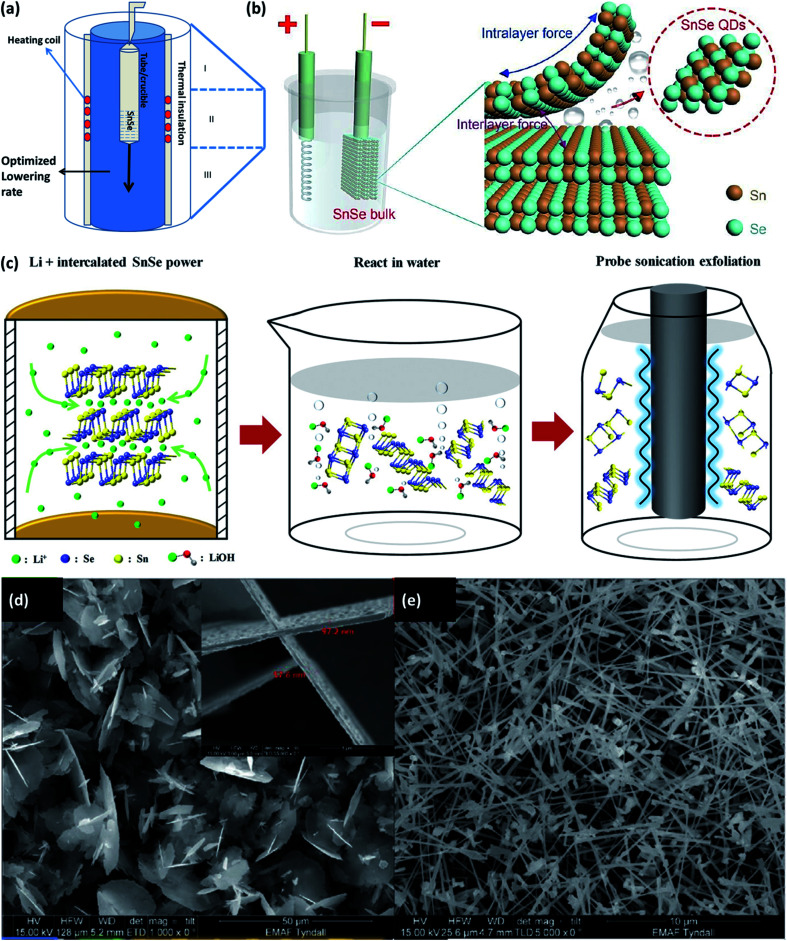
(a) Bridgeman–Stockbarger technique to grow a single crystal. (b) Schematic illustration for the synthesis of SnSe QDs by cathodic exfoliation. This figure has been adapted/reproduced from ref. 52 with permission from RSC, copyright 2019" (c) schematic synthesis process for the SnSe nanosheets by the liquid-phase exfoliation method includes two main steps: Li^±^ hydrothermal intercalation and sonication-assisted exfoliation. This figure has been adapted/reproduced from ref. 54 with permission from Wiley, copyright 2020". SEM images showing the evolution of the SnSe_*x*_ nanostructures concerning the growth temperature, (d) large flakes with diameters of several microns grown at 450 °C, with a thickness of ∼60 nm shown in the inset, (e) high yield of nanowire growth at 500 °C. This figure has been adapted/reproduced from ref. 49 with permission from Wiley, copyright 2020".

Bulk crystals of the SnSe and SnSe_2_ can be easily obtained by the physical solid-state reaction method. >99.99 pure elemental powders of the Sn and Se (in the stoichiometric ratio) is taken and sealed in the quartz tube at the pressure of ∼10^−4^ torr. It is placed at the temperature according to the Sn–Se system phase diagram, as shown in Fig. 2(a). The Bridgman method can be used to obtain single crystals. The powder was melted into the furnace and brought in the contacts with the single crystal's seed during its cooling. The material grows along the crystallographic orientation of the seed crystal. Depending upon the geometry it is called the horizontal Bridgman method or vertical Bridgman method. The Bridgman–Stockbarger technique as shown in (Fig. 2(a)) can be used to obtain a single crystal of SnSe and SnSe_2_ with an optimized ampule lowering rate of 7 mm h^−1^ and 4 mm h^−1^, respectively, for SnSe and SnSe_2_ with an optimized temperature gradient of 15 °C cm^−1^.^50^ Another technique to grow single crystals are the direct vapor transport method,^42^ and temperature gradient method.^51^ A detailed review of the growth of the SnSe single crystals can be found in an earlier study.^25^ Various methods to grow the nanostructure of the SnSe and SnSe_2_ is described in this section.

### 3.1 Synthesis of nanostructured SnSe

Li *et al.*^52^ devised the facile cathodic exfoliation method to exfoliate SnSe bulk into quantum dots with high yield. Under the organic electrolyte containing 0.2 M of tetra-butylammonium (at −7.5 V for 30 min) bulk SnSe was exfoliated and downsized up to ∼10 nm in lateral size, which was further downsized to ∼4 nm by breaking weakly entangled dots with the help of sonication as shown in Fig. 2(b). To synthesize the SnSe flake up to one layer, Jiang *et al.*^53^ reported the two-step process, in which first the bulk SnSe flakes were obtained by the atmospheric pressure vapor transport deposition method. Flash evaporation of SnSe powder was done by moving the quartz tube position containing SnSe powder at 700 °C under Ar/H_2_ gas pressure under atmospheric condition. SnSe is deposited on the Si substrate kept in upside-down position. As obtained flakes were etched with the N_2_ gas. Etching time of 5–20 min gave the single layer SnSe flake (thickness of 6.8 Å).^53^ The SnSe nanosheet of the best quality can be produced from the SnSe bulk powder by the three-step method.^54^ The method comprises the intercalation of Li^+^ ion into the SnSe layers by lithification and then reacting the material with water. The rapid expansion of H_2_ gas during reaction with water exfoliated the SnSe nanosheets. To improve the nanosheets' yield, further sonification of the SnSe powders' residuals is carried out (shown in Fig. 2(c)).

Using the precursor diselenoether SnCl_4_[BuSe(CH_2_)_3_Se^*n*^SeBu]}, SnSe_*x*_ nanostructures are formed on the Si(100) substrate coated with Au (acted as a catalyst) by the liquid injection chemical vapor deposition method, and growth was studied in the temperature range 450–550 °C. Under the 1.1 sccm flow of Ar gas and 1.5 mL h^−1^ of precursor injection rate, SnSe_2_ flake growth occurred at 450 °C, and considerable growth of SnSe nanowires occurred at 500 °C as shown in Fig. 2(d) and (e).^49^

### 3.2 Synthesis of nanostructured SnSe_2_

Choi *et al.*^55^ synthesized the nano-plates of the SnSe_2_ by dissolving 5 mL oleylamine in 50 mg, 0.26 mmol SnCl_2_ at 220 °C.

**Fig. 3:**
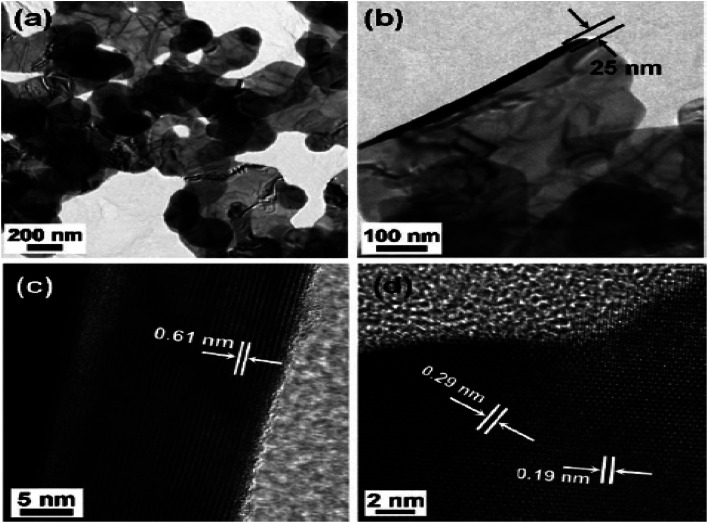
TEM image (a) and (b) SnSe_2_ nanoplates and high resolution TEM (HRTEM) images (c) side, and (d) top views. This figure has been adapted/reproduced from ref. 55 with permission from RSC, copyright 2011".

After that, a solution containing 1,3-dimethylimidazoline-2-selenone (90 mg, 0.53 mmol), dichloromethane (4 mL), and oleylamine (2 mL) were mixed and heated for 2 h at this temperature. After that, it was cooled to room temperature, centrifuged, and the residue was washed using hexane to obtain SnSe_2_ nanoplates. The transmission electron microscopy (TEM) image of SnSe_2_ nanoplates are shown in Fig. 3(a)–(d).

**Fig. 4:**
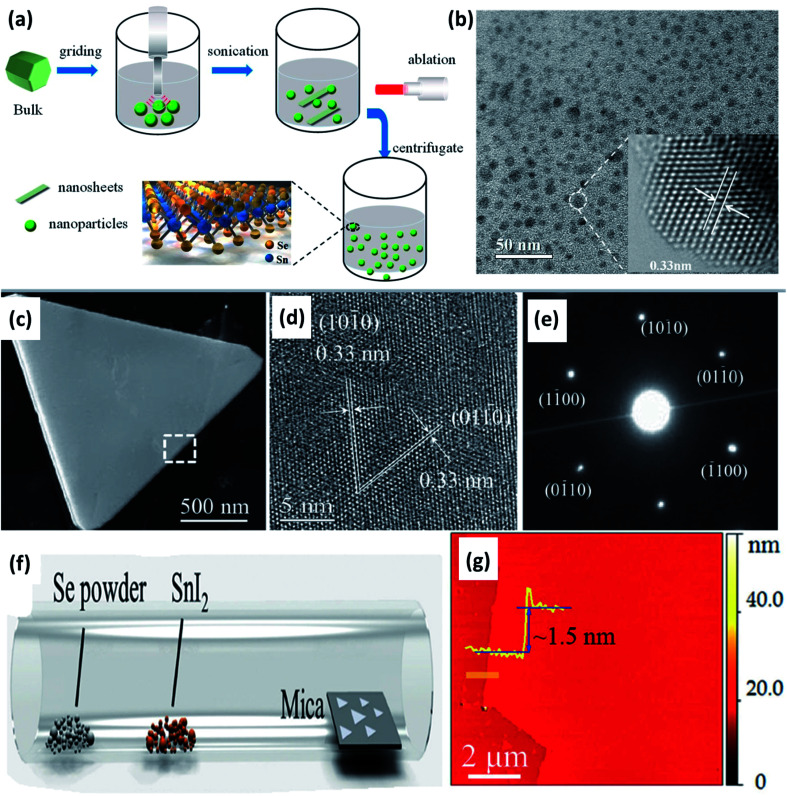
(a) Schematic show of the SnSe_2_ structure and the quantum dot (QD) fabrication process, and (b) TEM image of SnSe_2_ QDs with a centrifugal speed of 6000 rpm. These figures has been adapted/reproduced from ref. 56 with permission from MDPI, copyright 2019". (c) Low-magnification TEM image of a SnSe_2_ flake, (d) corresponding HRTEM image of the flake, (e) electron diffraction pattern from the same flake, (f) schematic diagram of the chemical vapor deposition method, and (g) a typical atomic force microscope (AFM) image at the flake edge, and the height profile showing a thickness of ∼1.5 nm. These figures has been adapted/reproduced from ref. 57 with permission from Wiley, copyright 2015".

A simple two-step method, *i.e.*, sonication followed by laser ablation, was used by Li *et al.* to obtain SnSe_2_ quantum dots (QD) from bulk powder.^56^ Manually grinded SnSe_2_ powder (20 mg) was dispersed in 30 mL deionized water, and the solution was sonicated (650 W) for 2 h in an ice bath for 4 s. Sonication resulted in smaller particles and flakes of SnSe_2_. The obtained solution kept in quartz cuvette was irradiated with a 1064 nm laser (Nd:YAG, 2.2 W) for 10 min. Irradiated tiny particles were centrifuged for 30 min at 6000 rpm, resulting in SnSe_2_ QD, as shown in Fig. 4(a) and (b).

Ma *et al.*^58^ synthesized the nanorods and nanoplates of the SnSe_2_ by organic solution phase route. Two different solutions of 0.4 mmol SnCl_2_·2H_2_O, 2 mmol oleic acid, 8.5 mL liquid paraffin oil, and 0.4 mmol selenium powder, 9 mL liquid paraffin were used. The first solution was heated at 160 °C till lemon yellow color appeared, and the later solution was heated at 240 °C till wine color appeared. The latter solution was injected into first using syringe rapidly and the mixture was heated at 200 °C for 20 min. After cooling, toluene and methanol was added. After centrifugation nanorods and nanoplates' were obtained. Chemical vapor deposition was employed by Zhau *et al.* to synthesize ultrathin (1.5 nm) SnSe_2_ flakes of high quality.^57^ Low melting point precursor SnI_2_ (0.01 g) and Se (0.1 g) powders were placed in two different alumina crucible, and mica substrate were placed ∼15 cm from the central zone. The central zone was heated at 600 °C for 15 min, with a flow of H_2_ (5 sccm) and Ar (20 sccm) at ambient pressure. As-synthesized flakes are shown in Fig. 4(c)–(g).

## 4. Various applications

**Fig. 5:**
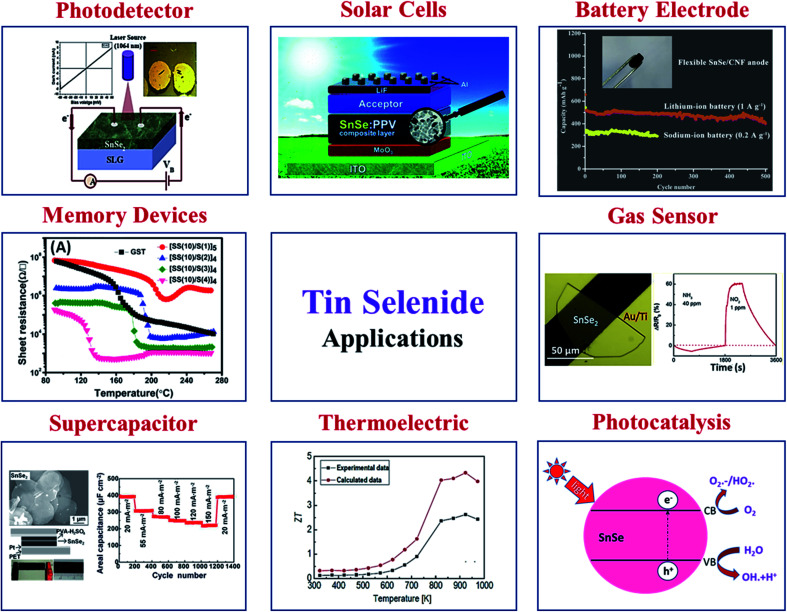
Various applications are based on SnSe materials. Solar cell, this figure has been adapted/reproduced from ref. 59 with permission from ACS, copyright 2010". Battery electrode, this figure has been adapted/reproduced from ref. 60 with permission from Elsevier, copyright 2020". Gas sensor, this figure has been adapted/reproduced from ref. 61 with permission from ACS, copyright 2019". Photocatalysis, this figure has been adapted/reproduced from ref. 62 with permission from Scielo, copyright 2017". Thermoelectric, this figure has been adapted/reproduced from ref. 38 with permission from Elsevier, copyright 2018". Supercapacitor, this figure has been adapted/reproduced from ref. 63 with permission from ACS, copyright 2014". Memory devices, this figure has been adapted/reproduced from ref. 64 with permission from AIP, copyright 2014". And photodetector, this figure has been adapted/reproduced from ref. 65 with permission from Elsevier, copyright 2020".

In recent times, tin selenide has gained significant research attention for solar cells, thermoelectric, photodetectors, photocatalytic, phase change memory, gas sensing, and anode material for battery, supercapacitor, topological insulator *etc.* Various reported applications based on SnSe materials are displayed in Fig. 5.

### 4.1 Thin film solar cells (TFSCs)

Fabrication of low-cost thin-film solar cells is highly needed as they can also be deposited over flexible substrates. The CuInGaSe_2_ (CIGSe) and CdTe based solar cells have shown record small cell power conversion efficiency (PCE) of 23.35% and 22.1%, respectively.^66^ But in CIGS TFSC, indium is rare, and gallium is very costly. In CdTe TFSC, Cd is toxic, and Te is a rare material. These materials related problems hinder the commercialization of low-cost, earth-abundant TFSC based on CISG and CdTe. In recent time, earth-abundant and low-cost compound semiconductor materials like Cu_2_ZnSnS_4_ (CZTS), Cu_2_ZnSnSe_4_ (CZTSe), and Cu_2_ZnSn(S,Se)_4_ (CZTSSe) have gained massive consideration as alternate materials instead of CIGS and CdTe as they have shown the efficiency of 10%, 11.95%, and 12.62%, respectively.^67–69^ But, kesterite material consists of many elements.^70^ The presence of many elements in the absorber layer increases the processing cost, and defects are also formed during the processing, which ultimately reduces the overall PCE of solar cells. To overcome this problem, binary element-based materials with similar optical and electronic properties are more suited than quaternary element-based kesterite materials. Tin sulfide (SnSe) is also attracting attention of researchers for optoelectronic device applications. In SnSe, only one impurity phase of SnSe_2_ is present.^71^ Therefore, the formation of phase SnSe is easier. Also, being consists of earth-abundant, inexpensive, and eco-friendly elements, SnSe has attracted significant attention. The SnSe has an optimum bandgap of 1.1 to 1.3 eV, an absorption coefficient of ∼10^5^ cm^−2^, and p-type conductivity with high carrier concentration (10^17^ cm^−3^), making it a fabulous material for solar photovoltaic applications.^8^ Due to its optimum bandgap (1.3 eV) and high absorption coefficient (10^5^ cm^−1^), a thin layer of 300 nm thickness can absorb most of the useful solar spectrum. The tunable bandgap of thin-film SnSe can help absorb more solar radiation and enable more photon absorption, resulting in more electron–hole pair generation. The theoretical efficiency for material with a bandgap of 1.3 eV is 32%.^72^ The SnSe thin film can be deposited using various chemical and physical routes like chemical bath deposition (CBD),^73^ electrodeposition,^74^ spray pyrolysis,^75^ atomic layer deposition (ALD),^17^ thermal evaporation,^46^ sputtering.^76,77^

The device architecture for SnSe TFSC is SLG/Mo/SnSe/CdS/i-ZnO/TCO/metal grid, similar to CIGS,^78^ and CZTS/CZTSe/CZTSSe TFSCs.^79^ Therefore, a lot of modifications and optimizations are required. Also, many issues need to be mitigated to improve efficiency.^80^ Consequently, it is essential to conduct a study on SnSe material to develop environmentally-friendly, low-cost TFSCs. Any photovoltaic materials should have good optical properties, appropriate band gap, good absorption coefficient, high carrier concentration, and better transport properties. Solar cell efficiency may get affected due to losses, *i.e.*, optical losses, non-absorption, thermalization, reflection loss, transmission loss, area loss, collection losses, and resistance losses.^81^ However, the formation of pure phase SnSe thin films has not been reported as the phase lies in a very narrow region, and fine compositional tuning is required to achieve SnSe pure phase. Sn and Se's reaction mechanism plays a crucial role in attaining pure phase SnSe thin films.^19,82^ Reddy *et al.* studied the effect of selenization temperature, elemental composition, and selenization pressure on SnSe. They observed that the single-phase SnSe thin films could be achieved during the selenization process in the temperature ranges from 300 to 500 °C.^77^ The solar cell efficiency depends on various parameters, like deposition condition, the absorber's crystallinity, carrier concentration, and the p–n junction's nature. The lower solar cell efficiency based on SnSe thin films may be due to SnSe absorber materials' low quality. The parameters that highly influence SnSe thin film quality are phase purity, higher crystallinity, and larger grain size with fewer pinholes. It has been observed that during SnSe phase formation, there is a possibility of the formation of the SnSe_2_ phase. Thus, the SnSe_2_ phase's impact on the solar cell performance of SnSe thin films needs to be carefully studied and optimized.^8^ The electronic properties of p–n junction interfaces are strongly influenced by the discontinuities between the valence band (VB) maxima and conduction band (CB) minima of each material, which restrict the electron transport across the junction interfaces. Band alignment of SnSe with an n-type buffer layer, the density of defect states at the hetero-junction interface, and the nature of back contact also influence the solar cell performance. Therefore, band offsets play a crucial role in reducing the dark current in a diode, reducing the photon-generated carrier losses, and improving the overall solar cell conversion efficiency. The n-type buffer layer with a wider band gap is needed to overcome the optical losses and provide better alignment with SnSe absorber material.

In 1990, Singh *et al.* reported SnSe based solar cells with power conversion efficiency (PCE) of 2.3%.^75^ The fabricated solar cell showed an open-circuit voltage (*V*_oc_) of 410 mV, short-circuit current density (*J*_sc_) of 9.20 mA cm^−2^, and a fill factor (FF) of 49%, respectively. Rahman *et al.* fabricated heterojunction (p-SnSe/n-Si) solar cells and achieved an efficiency of about 6.44%.^83^ They observed improved power conversion efficiency due to the improvement in the *J*_sc_ with a graded junction.^83^ Shinde *et al.* fabricated SnSe thin-film solar cells using electro-deposition and exhibited device efficiency of 1.4% using an absorber film with 300–400 nm-sized grains, uniform, and dense film morphology.^73^ Franzman *et al.* synthesized SnSe/PPV (poly[2-methoxy-5-(3′,7′-dimethyl octyl oxy)-1,4-phenylenevinylene]) nanocrystals for solar cells and observed improvement in efficiency from 0.03% to 0.06%.

**Fig. 6:**
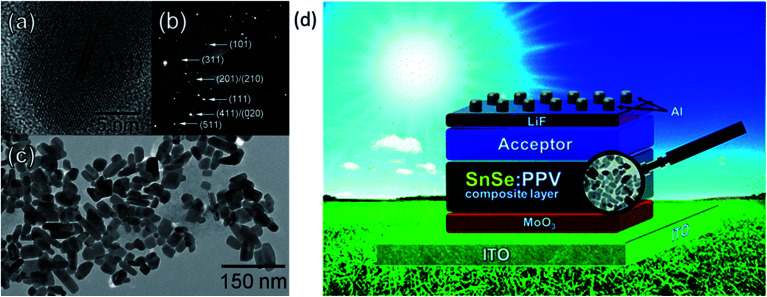
(a) HRTEM image of a single nanocrystal. (b) Selected area electron diffraction (SAED) pattern of SnSe. (c) Low-resolution TEM image of SnSe nanocrystals. (d) The device structure of SnSe solar cells. These figures has been adapted/reproduced from ref. 59 with permission from ACS, copyright 2010".

They observed significant improvement in external quantum efficiency (EQE) and *J*_sc_ after adding PPV into the SnSe absorber. Fig. 6(a) and (b) show the high-resolution transmission electron microscopy (HRTEM) image of a single nanocrystal, and selected area electron diffraction (SAED) pattern of SnSe. Fig. 6(c) shows a low-resolution TEM image of SnSe nanocrystals, and Fig. 6(d) shows the schematic device structure of SnSe solar cells.^59^ In 2012, Mathews *et al.* reported the first inorganic thin-film solar cell and exhibited a device efficiency of 0.03% (*V*_oc_ = 140 mV, *J*_sc_ = 0.7 mA cm^−2^). The low device efficiency is mainly due to poor junction quality.^74^ Makori *et al.*^84^ fabricated solar cell with device structure glass/Ag/CdO:Sn/SnSe/Ag by using thermal evaporation and reported device efficiency of 0.59% (*V*_oc_ = 273 mV, *J*_sc_ = 0.993 mA, and FF = 69%). The observed lower efficiency is mainly due to the thin absorber (148 nm) layer, which attributed to lower *J*_sc_ in the device. The above discussion is summarized in Table 1.

**Table 1:** Summary of SnSe based thin-film solar cells

Deposition method	Absorber	Device architecture	*V*_oc_ (mV)	*J*_sc_ (mA cm^−2^)	FF (%)	*η* (%)	Area (cm^2^)	Ref.
Evaporation	SnSe	Si/Al/SnSe/In	425	17.23	44	6.44	0.2	83
Evaporation	SnSe	SLG/FTO/Se/SnSe/Ag	410	9.20	49	2.30	—	75
Electro-deposition	SnSe	ITO/CdS/SnSe/Au	370	5.37	30	1.40	—	73
Co-evaporation	SnSe	SLG/Mo/SnSe/CdS/	172	18.87	31.2	1.02	0.42	10
		i-ZnO/ITO/Ni–Al						
Sputtering	SnSe	Glass/Ag/CdO:Sn/SnSe/Ag	207	0.90	0.69	0.59	—	84
Electrodeposition	SnSe	Tec15/CdS/SnSe/carbon-paste	140	0.7	31	0.03	0.15	74

Table 1 shows that the efficiency of SnSe based solar cells is well below Cu_2_ZnSn(S,Se)_4_.^85^ The observed lower efficiency is mainly due to bulk defects, secondary phase formation in SnSe, non-ideal band-alignment at heterojunction interfaces, and back and front electrode recombination. The absorber quality plays a significant role, and a sound absorber should have a dense, pinhole-free, smooth surface of the film for high-efficiency heterojunction device applications. The low interfacial quality could primarily affect the device performance and is responsible for lower *V*_oc_ and FF in the solar cells. This is mainly due to CB discontinuity/band offsets at the interfaces, which leads to the CB's energy barrier and restricts carrier transportation across the p–n junction. Apart from this, absorber thickness, grain size, bandgap, and deposition methods also influence the device performance.

### 4.2 Thermoelectric generators

It requires energy to maintain the sustainability and development of humankind. Energy sources are minimal, and there arises a question about the world's power crisis and its ultimate solution. A thermoelectric device that can convert the waste heat into electricity can contribute to a remarkable extent in this.

#### 4.2.1 SnSe thermoelectric

##### SnSe single crystal

With the report of a high *ZT* value of 2.6 along the *b* axis in single-crystal SnSe,^32^ this material became the hot topic of research in the field of thermoelectric (TE) domains since 2014. SnSe is anisotropic and exhibits the second-order displacive phase transition from *Pnma* (distorted) to *Cmcm* (non-distorted) at 750–800 K. It shows a moderate power factor of 10.1 μW cm^−1^ K^−2^ at ∼850 K along the *b* axis, which is comparable or even lesser than typical thermoelectric materials values.^86–89^ The thermal conductivity of SnSe has astonished the researchers. Using the Gruneisen parameter, it has been estimated that SnSe exhibits the ultra-low thermal conductivity (<0.25 W m^−1^ K^−1^) due to the strong anharmonicity in bonding.^32^ Theoretical studies^90,91^ showed that optimized carrier concentration lies in the range 10^19^–10^20^ cm ^−3^ for SnSe for high *ZT*. For n-type SnSe, the estimated *ZT* is 3.1 along *a* axis at 770 K at carrier concentration of 2.8 × 10^19^ cm^−3^, while the study showed that the n-type SnSe performed better than p-type SnSe.^92^

**Fig. 7:**
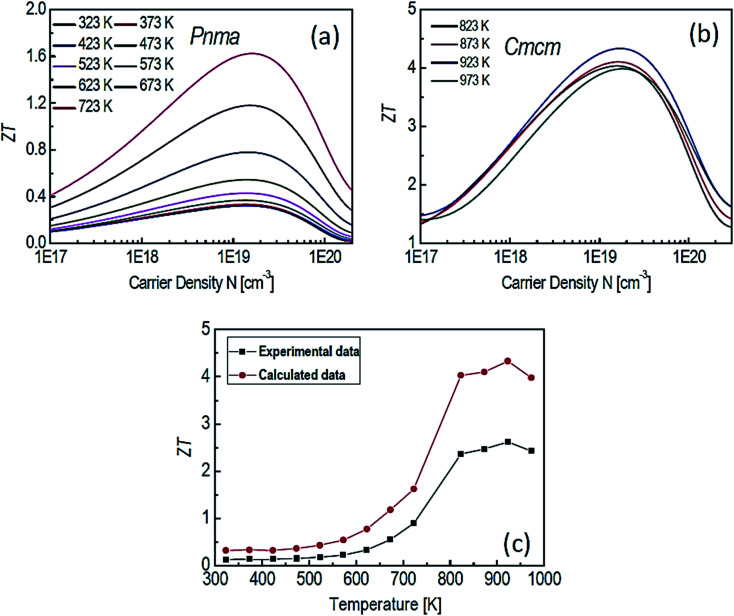
The *ZT* of SnSe for *Pnma* (a), *Cmcm* (b) phases and comparison between experimental data^32^ and calculated data (c). These figures has been adapted/reproduced^38^ with permission from Elsevier, copyright 2018".

Motivated by the high *ZT* along the *b* axis in SnSe, Li *et al.*^38^ conducted the theoretical study on the p-type SnSe using the first-principle calculation. A figure of merit (*ZT*) was greatly affected by the carrier concentration and also sensitive to the phases of SnSe (*Pnma* and *Cmcm*) (shown in Fig. 7(a) and (b)). Fig. 7(c) shows a comparison of theoretical and experimental data.^32^ This study showed that a maximum *ZT* of 4.33 could be achieved for the *Cmcm* phase of SnSe at 923 K for carrier concentration of 1.84 × 10^19^ cm^−3^, along the *b* axis.

Through another study, for Na doped SnSe single crystal, Zhao *et al.* reported the device with *ZT*_device_ = 1.34 in the temperature range 300–773 K. With *ZT*_max._ of 2.0 at 773 K, the device showed the conversion efficiency of ∼16.7%, which was higher than many Pb-based thermo-electric modules.^93^ Various researchers showed different *ZT* values for the single crystal. For example, Wei *et al.*^94^ showed *ZT* of ∼1, ∼0.8, and ∼0.25 along *b*, *c*, and *a* axis, respectively for in-house made fully dense single-crystalline SnSe, Jin *et al.*^95^ showed *ZT* ∼ 1 for single crystal grown by a vertical vapor deposition method. Different types of doping are being done to enhance the single-crystal performance. Chang *et al.*^96^ doped bromine to make the SnSe conduction n-type and achieved record high *ZT* ∼ 2.8 ± 0.5 at 773 K along out of the plane direction. With the help of density functional theory and scanning tunneling microscopy, they pointed out that delocalized p electrons of Sn and Se near conduction band minima contribute more to orbital overlapping in out of the plane direction. The S doping in single-crystal SnSe decreased the carrier concentration due to charge trapping at grain interface.^97^ Hence, S doping decreased the single crystal's thermoelectric performance rather than enhancing as predicted by theoretical calculation.^98^ Pb doped n-type single crystal (*via* facile Sn-flux method) showed a 33% enhanced power factor than its pristine SnSe due to increased carrier concentration.^99^ Ag-doped single crystal (Sn_0.97_Ag_0.03_Se) grown in a horizontal Bridgman furnace showed maximum *ZT* ∼ 0.95 along *a* axis at 793 K.^100^ Bi doping in single-crystal SnSe resulted in n-type conduction and showed the remarkable *ZT* ∼ 2.2 along the *b* axis at 733 K.^51^

##### Polycrystalline SnSe thermoelectric

Polycrystalline SnSe has comparably higher thermal conductivity and lower electrical conductivity than its single crystal counterpart. Though phonon scattering is reduced mainly by the grain boundaries,^101^ it has higher thermal conductivity than its single crystal due to tin oxide layer^102^ or absorption of oxygen.^103^ Highly discrete values of *ZT* have been reported for polycrystal SnSe made by different growth techniques. Various researchers have tried to achieve a comparable result to a single-crystal. Many researchers chose to dope (Na, K, Cu, Zn, *etc.*) in poly-crystal SnSe to enhance the electrical properties and reduce the thermal conductivity effectively. In one study, the polycrystalline SnSe thermoelectric material showed *ZT* ∼ 0.5 at 823 K.^104^ Several methods improve the device's thermoelectric performance, like large mass fluctuations, band gap engineering, alloying, doping, nano-structuring, *etc.*^105^ Gong *et al.*^47^ studied the effect of Cu doping in polycrystalline SnSe by varying the concentrations in the Cu_*x*_Sn_1−*x*_Se from *x* = 0.01 to 0.004. They found that Cu doping enhanced the electrical conductivity due to the increased carrier concentration, as confirmed by Hall measurement. Still, Cu doping acted as a point defect and hence decreased the carrier mobility. The Seebeck coefficient also increased due to doping. Thus, the overall power factor increased due to doping, and the optimized value was obtained for doping *x* = 0.01. Around 60% reductions in the lattice thermal conductivity as compared to undoped poly-crystal SnSe is observed. This was due to nano-precipitation and mesoscale grains (as evidenced by high-resolution transmission electron microscopy (HRTEM)). Finally, they achieved a *ZT* of 1.2 at 873 K. Shi *et al.*^106^ enhanced the Cu's solubility limit in the SnSe and obtained the *ZT* of ∼1.41 for the doping level of *x* = 0.118.

**Fig. 8:**
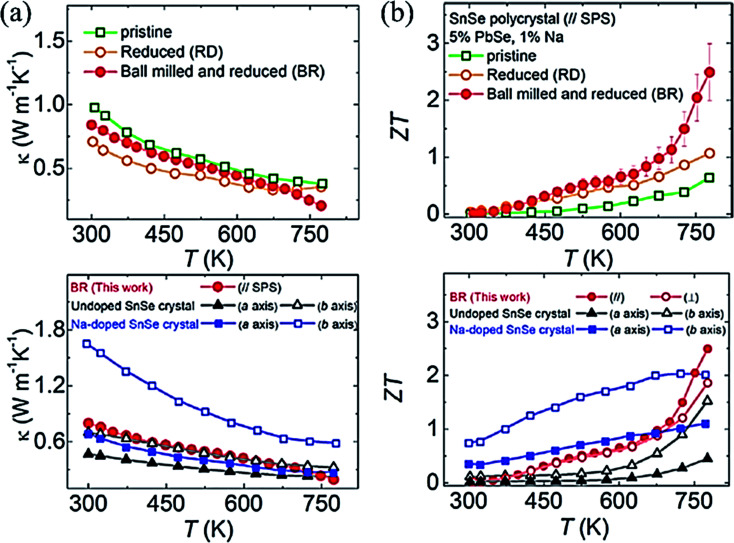
(a) Total thermal conductivity of polycrystalline SnSe–PbSe (5 mol %) doped with 1 mol% Na for pristine, Reduced (R), and Ball milled then Reduced (BR) samples taken parallel to the press direction of SPS, and total thermal conductivity compared with undoped SnSe^32^ and Na doped SnSe,^93^ and (b) ZT of polycrystalline SnSe–PbSe (5 mol %) doped with 1 mol% Na for pristine, Reduced (R), and Ball milled then Reduced (BR), and ZT compared with the undoped SnSe^32^ and Na doped SnSe.^93^. These figures has been adapted/reproduced from ref. 102 with permission from Elsevier, copyright 2019".

Lee *et al.*^102^ synthesized polycrystalline SnSe (Na_0.01_(Sn_0.95_Pb_005_)_0.99_Se), which exhibited *ZT* ∼ 2.5 at 773 K. They noticed that the oxide formation leads to higher thermal conductivity in poly-crystal SnSe (SnO_2_ has ∼140 times higher thermal conductivity than SnSe).^107^ They removed the oxides layers of the tin in SnSe by Ball milling followed by a reduction in 4% H_2_/Ar atmosphere for 6 h at 613 K. They pointed out that ball milling (BM) and reduction processes both are critically important to remove the oxides and its residuals from the sample. A remarkably ultralow thermal conductivity (total) of 0.20 W m^−1^ K^−1^ (even lower than the single crystal reported by Zhao *et al.*^32^ ) (Fig. 8(a)) and ∼2 times enhanced power factor than the pristine SnSe (unball-milled and unreduced). The performance of pristine, reduced, ball-milled and reduced (BR) compared to with the single-crystal SnSe^32^ and Na doped SnSe,^93^ is shown in Fig. 8(b). Recently Gainza *et al.*^108^ reported the highest *ZT* of 1.8 for the un-doped polycrystalline SnSe, and even with surface oxides layers on the sample.

The pressure-induced effect modifies the textures of the poly-crystal SnSe. A sintering pressure of 60 MPa leads to improved electrical properties. There was a meager improvement in thermal conductivity, resulting in a *ZT* of 0.7.^109^ There is a vast literature on doping in the polycrystalline SnSe, like Na,^110^ K,^111^ Cd,^112^ Ag,^113^ and co-doping Na/Ag,^114,115^ Bi/Cl,^116^ Na/CNT,^117^*etc.* which resulted in the enhancement in the thermoelectric performance of the material. Lu *et al.*^118^ enhanced the performance of polycrystalline SnSe by introducing large mass fluctuations by doping sulfur, which led to very low lattice thermal conductivity (0.13 W m^−1^ K^−1^ at 873 K). Still, this doping enhanced the bandgap of the SnSe, which lowered the electrical conduction and hence the power factor (low carrier concentration due to large bandgap). Pb co-doping improved the power factor, further increasing the carrier concentration by one order of magnitude. Through co-doping a remarkable *ZT* of 1.85 at 873 K was achieved. The inclusion of micro carbon fibers into the polycrystalline SnSe decoupled the thermal and electrical transport in the host matrix. Carbon fibers acted as good electrical conductors and simultaneously reduced the lattice thermal conductivity (0.22 W m^−1^ K^−1^) by enhancing the scattering due to the high density of interface, which led to *ZT* ∼ 1.3 at 823 K. This inclusion increased the mechanical stability of the device.^119^ The thermoelectric parameters of poly-crystals SnSe are tuned by sintering temperature in the spark plasma sintering (SPS). Zhang *et al.*^120^ varied the sintering temperature from 300 to 650 °C in SPS and showed that vacancy defects (Se, Sn, and Se–Sn) were responsible for the performance variation.

**Fig. 9:**
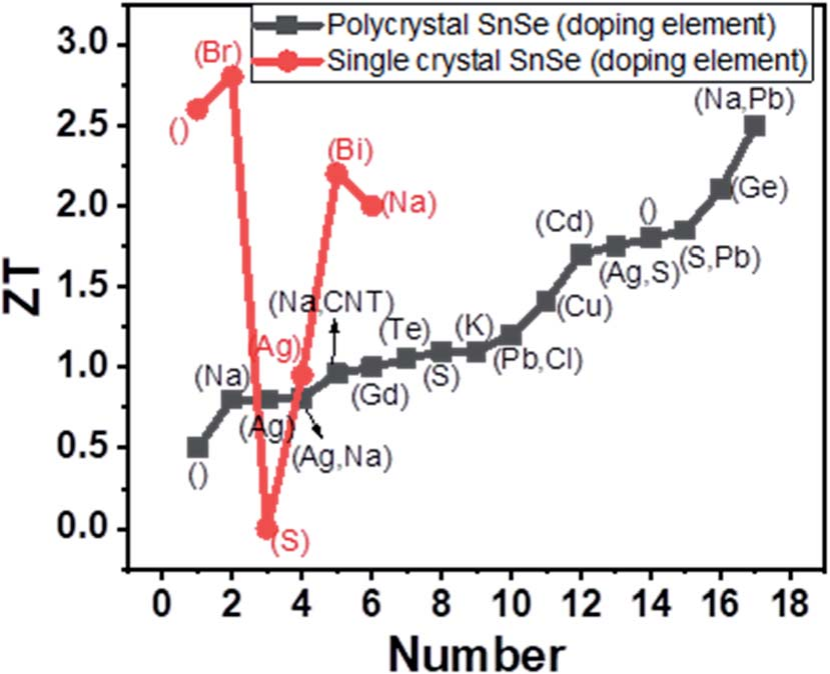
Variation of *ZT* with the doping element given in bracket, where blank () shows the undoped SnSe.

The optimized sintering temperature for the best performance of the material was 550 °C. The *ZT* of 0.47 was observed at 430 °C. Ge alloying's effect was studied by varying Ge concentration in the range *x* = 0.01 to 0.03 (Sn_1−*x*_Ge_*x*_Se). The carrier concentration increased from 3.9 × 10^17^ to 4.2 × 10^19^ cm^−3^ for *x* = 0.03, which resulted in a high-power factor of ∼5.10 μW cm^−1^ K^−2^ at 873 K. A very low lattice thermal conductivity of ∼0.18 W m^−1^ K^−1^ was achieved due to nanoscale grains, precipitation and anharmonicity due to Ge doping. Finally, high *ZT* of 1.75 and 2.1 were acquired at 873 K along parallel and perpendicular SPS's pressing direction.^121^ The figure of merit (*ZT*) with different doping elements in single and polycrystal SnSe is shown in Fig. 9.

##### SnSe thin film

Low temperature (4–300 K) thermoelectric measurements were carried out by Urmila *et al.*^122^ in SnSe thin-film obtained by the co-evaporation method. The maximum power factor obtained was 7.2 × 10^−4^ W m^−1^ K^−2^. The thermal conductivity was in the range of 0.023 to 0.045 W m^−1^ K^−1^, which showed a maximum *ZT* of 1.2 at 42 K, and thus showed a potential application in a low-temperature TE device. Nair *et al.*^123^ fabricated the n–p type thermo-couples with PbSe (p-type), SnSe (p-type), and SnSe_2_ (n-type), in the configuration SnSe–SnSe_2_–SnSe–SnSe_2_–SnSe and PbSe–SnSe_2_–PbSe–SnSe_2_–PbSe using chemical method. A voltage was developed for a temperature difference of 20 °C of magnitute 50 mV and 15 mV, respectively (the author did not report the *ZT* of the device). Thin-film SnSe deposited by glancing angle (80°) pulsed laser revealed a striking power factor of 18.5 μW cm^−1^ K^−2^ at 478 K (higher than the single crystal along *b* axis) due to enhanced grain boundaries and out of plane thermal conductivity (highest 0.189 W m^−1^ K^−1^ at 340 K). The authors did not report the *ZT* and in-plane thermal conductivity.^124^ For the first time, Burton *et al.*^125^ reported thin film SnSe thermoelectric generator on the glass substrate. Very low thermal conductivity of 0.08 W m^−1^ K^−1^ in the temperature range 375 to 450 K was observed (4 times lower than single-crystal thermal conductivity along *a*-axis). Still, it had a low power factor, which resulted in a very low *ZT* of 0.055 at 501 K. When the hot side was kept at 618 K, an output of 0.09 μW was achieved. Due to the oxide formation and uncontrolled cold side temperature, the performance degraded for a longer exposure. Song *et al.*^126^ synthesized SnSe thin film of varied thickness in the range of 600–1000 nm by magnetron sputtering and showed effect of post-annealing on the performance. The optimized annealing temperature was 700 K. The power factor for the film with thickness 700 nm was 2.4 μW cm^−1^ K^−2^ at 675 K. The authors used the thermal conductivity data of bulk polycrystalline SnSe to calculate the *ZT* (0.28 at 675 K). Yan *et al.*^127^ fabricated a flexible thermoelectric device based on SnSe composite with PEDOT:PSS that showed 13.73 nW output power at 50 K temperature difference at 353 K, and showed possible application in wearable electronics as well due to stability of device even after 1000 cycles of bending. The above discussion is summarized in Table 2.

**Table 2:** Thermoelectric properties of SnSe based materials

Material	Synthesis method	Power factor (μW (cm^−1^ K^−2^))	Thermal conductivity (W m^−1^ K^−1^)	Temp. (K)	*ZT*	Ref.
Pristine SnSe (single crystal)	Bridgman Crystal growth	∼9	0.34	923	2.62	32
Single crystal	Vertical Bridgman	8.5	—	850	1	128
Single crystal	Vertical vapor deposition	5.23	0.44	800	1	95
Na doped SnSe single crystal	—	∼14	∼0.55	773	∼2	93
Br doped SnSe single crystal	Temperature gradient	∼9	∼0.23	773	2.8	96
S doped single crystal	Direct vapor transport	0.0011	—	573	0.0012	97
Pb doped SnSe single crystal	Flux method	1.2	—	300	—	99
Ag-Doped SnSe single crystal	Horizontal Bridgman method	∼5.7	0.49	793	0.95	100
Bi-Doped SnSe single crystal	Temperature gradient	∼9.75	∼0.3	733	2.2	51
Polycrystal SnSe	Solid state reaction (SSR)	∼4	<0.5	823	0.5	129
Polycrystal SnSe	Arc melting	∼10	∼0.4	816	1.8	108
Cu doped polycrystal SnSe	Hydrothermal	3.4	0.26	873	1.2	47
SnSe_0.98_Te_0.02_	—	∼4	∼0.35	805	1.05	130
Sn_0.97_Gd_0.03_Se	Hydrothermal	6.7	0.4	868	∼1	131
Sn_0.985_S_0.25_Se_0.75_	Mechanical alloying	4.5	∼0.35	823	1.1	132
Sn_0.978_Ag_0.007_S_0.25_Se_0.75_		5.3	∼0.25	823	1.75	
Sn_0.90_Pb_0.15_Se_0.95_Cl_0.05_	SSR	6.74	0.5	823	1.2	133
Cu doped polycrystal SnSe	Solvothermal	5.57	0.32	823	1.41	106
Na, Pb doped polycrystal SnSe	SSR	6.85	0.20	773	2.5	134
Polycrystal SnSe	Mechanical alloying	3.9	—	823	0.7	109
Na doped SnSe polycrystal	SSR	4.5	∼0.4	773	0.8	110
K doped SnSe polycrystal	Mechanical alloying	∼2.9	∼0.22	773	1.1	111
Cd doped SnSe polycrystal	Solvothermal	6.9	0.33	823	1.7	112
Ag doped polycrystal SnSe	Facile surfactant-free synthesis	6.34	0.75	850	0.8	113
Ag/Na doped polycrystal SnSe	—	∼7.5	∼0.5	773	1.33	114
Ag/Na doped polycrystal SnSe	SSR	5	∼0.48	773	0.81	115
CNT dispersed Na doped polycrystal SnSe	SSR	4.99	0.40	773	0.96	117
S, Pb doped polycrystal SnSe	Hydrothermal	4.18	∼0.20	873	1.85	118
Composite of carbon fiber and polycrystal SnSe	SSR	3.88	∼0.22	823	1.3	119
SnSe polycrystal	Solvothermal	∼4.4	∼0.7	703	0.47	120
Ge doped polycrystal SnSe	Hydrothermal	5.1	∼0.21	873	2.1	121
Thin film SnSe	Reactive evaporation	7.2	∼0.023	42	1.2	122
SnSe thin film	PLD	18.5	—	478	—	124
SnSe thin film	Thermal evaporation	0.11	∼0.11	501	0.055	125
SnSe thin film	Sputtering	2.4		675	0.28	126
SnSe thin film	Chemical vapor transport	—	0.7	300	0.16	135
SnSe thin film	Chemical vapor deposition	3.2	1.1	550	0.15	136
SnSe/PEDOT:PSS thin film	Vacuum filtration method	0.24	—	353	—	127

#### 4.2.2 SnSe_2_ thermoelectric

**Fig. 10:**
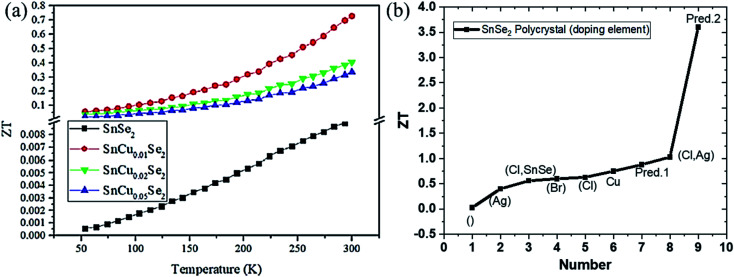
(a) Figure of merit (*ZT*) of SnCu_*x*_Se_2_ (*x* = 0, 0.01, 0.02, 0.05) as a function of temperature. This figure has been adapted/reproduced from ref. 145 with permission from ACS, copyright 2020". (b) Variation of *ZT* with different doping elements, irrespective of operating temperature, where blank () represents undoped SnSe_2_, and Pred. 1 and Pred. 2 mean theoretical predicted values, respectively.

A theoretical study on SnSe_2_ using the first principal method revealed its anisotropic nature. Due to the bipolar conduction nature (because of the low bandgap) optimal predicted doping values were (0.86–2.03) × 10^19^ and (1.71–2.47) × 10^19^ cm^−3^ along with the *a* and *c* axes, respectively. A maximum power factor of 11.72 × 10^−4^ W m K^−2^ was expected for a doping concentration of 7.21 × 10^19^ cm^−3^ at 800 K along *a* axis. Minimum thermal conductivity predicted along *a* and *c* axes at 300 K were 0.55 and 0.42 W m^−1^ K^−1^, respectively. Finally, *ZT* value was expected along *a* direction that showed a value of 0.88 for 1.94 × 10^19^ cm^−3^ at 800 K.^137^ Saha *et al.* synthesized the SnSe_2_ nanosheets using a solution-based method, and Cl doping was done to enhance the carrier concentration.^138^ At room temperature carrier concentration increased from 7 × 10^17^ to 2 × 10^18^ cm^−3^ due to Cl doping. For the doped sample, almost double power factor of 1.46 μW (cm^−1^ K^−2^) was achieved at 630 K. As synthesized SnSe_2_ nanosheets showed the thermal conductivity in the range of ∼0.45 to 0.35 W m^−1^ K^−1^ while doping increased the thermal conductivity in the range of 0.67 to 0.40 W m^−1^ K^−1^ at 300 to 630 K temperature, respectively. Overall, *ZT* of 0.22 was achieved at 610 K for Cl doped samples. For heavy Cl doping of *x* = 0.12 by solid-state reaction, *ZT* (out of plane) of ∼0.4 was achieved by Xu *et al.*^139^ Se deficiency and Cl doping's led to enhanced power factor and reduced thermal conductivity resulting in a *ZT* of 0.63 at 673 K along an in-plane direction.^140^ A study on Ag doping revealed that it effectively enhances the carrier mobility but decrease the carrier concentration. For 1% doping, the optimized power factor was 3.50 μW cm^−1^ K^−2^ at 773 K, which resulted in a *ZT* of ∼0.4.^141^ In another theoretical study, Ding *et al.* reinvestigated the thermoelectric performance of the SnSe_2_ by the first principle method. They considered various models of the phonon–phonon scattering, which may overestimate the thermal conductivity.^33^ Room temperature electrical conductivities along *a* and *c* directions were calculated as 4.97 × 10^5^ and 3.39 × 10^4^ Ω^−1^ m^−1^, respectively, at 10^20^ cm^−3^ carrier concentration. Overall, for n-type highly doped (10^20^ cm^−3^) SnSe_2_, a *ZT* of 3.6 could be achieved at 800 K. Wu *et al.* doped Br to the site of Se in polycrystalline SnSe_2_ to enable broad carrier concentrations (0.5 to 5.6 × 10^19^ cm^−3^, saturation at doping of *x* = 0.01) consistent with the single parabolic model.^142^ They reached a *ZT* of 0.6 at 750 K, which was competitive to polycrystalline SnSe. The Cl-doped composite of SnSe–SnSe_2_ showed *ZT* ∼ 0.56 at 773 K.^143^ Dynamical intercalation of Ag into the weak van der Waals interlayers of SnSe_2_ acted as electron donor and introduced line defect, twin boundary dislocation, phase interface, and enhanced phonon–phonon scattering. To further strengthen carrier concentration into Ag-doped SnSe_2_, Liu *et al.* doped it with Cl, which resulted in concentration in the range of 10^19^ due to Fermi leveling up to the conduction band (as shown *via* DFT calculation).^144^ Ag interlayer bridge weakened the anisotropy of electrical transport; hence the power factor along parallel and perpendicular directions of pressing were 7.46 and 8.25 μW cm^−1^ K^−^^2^, respectively, at 789 K, and a maximum *ZT* of ∼1.03 was achieved at 789 K along the parallel direction of the pressure. Recently, Wang *et al.* showed the room temperature thermoelectric potential of the SnSe_2_ embedded with Cu, which enhanced carrier concentration two orders of magnitude. Optimum doping in SnCu_*x*_Se_2_ was found to be *x* = 0.01, which resulted in an optimized power factor of 1.96 at room temperature and a decrease in thermal conductivity up to 0.81 W m^−1^ K^−1^ due to increased interface.^145^ A very high *ZT* of 0.75 than to pristine SnSe_2_ was obtained which was about two orders of magnitude larger than pristine SnSe_2_ (Fig. 10(a)).

##### Thin-film SnSe_2_ thermoelectric

Thermoelectric performance of SnSe_2_ thin films have also been studied. But there are very few reports on this, and the reports are limited to the film's power factor only. Yin *et al.* deposited SnSe_2_ thin-film on a glass substrate by spin coating techniques and reported a Seebeck coefficient of ∼126.3 μV K^−1^. The electrical conductivity of the order of 10^3^ S m^−1^ at room temperature was observed.^146^ Chen *et al.* deposited the SnSe thin film on the Si wafer and annealed it in Se vapor. The film was reduced to SnSe_2_ and showed enhanced Seebeck coefficient 631 μV K^−1^ for SnSe_2_ than 38.6 μV K^−1^ for SnSe. Around 44 times enhanced power factor (0.2 μW m^−1^ K^−^^2^) was obtained.^147^ Duong *et al.* deposited SnSe_2_ thin film on the Al_2_O_3_ substrate by the pulsed laser deposition technique. They reported a power factor of 8 μW m^−1^ K^−2^ at 220 K.^148^ A detailed summary of the above discussions and *ZT* variation with the doping is provided in Table 3 and Fig. 10(b), respectively.

**Table 3:** Thermoelectric properties of doped-SnSe_2_ based materials

Material	Synthesis method	Power factor (μW cm^−1^ K^−2^)	Thermal conductivity (W m^−1^ K^−1^)	Temp. (K)	*ZT*	Ref.
Cl doped SnSe_2_	Facile low-temperature solution	1.46	∼0.45	610	0.22	138
Cl doped SnSe_2_	Solid-state reaction followed by hot press	∼7.0	∼1.6	673	0.4	139
Cl doped SnSe_2_	Solid-state reaction followed by ball milling and SPS	∼8	∼1.2	673	0.63	140
Ag-Doped SnSe_2_	Mechanical alloying followed by SPS	3.50	<1	773	0.4	141
Br doped SnSe_2_	Solid-state reaction followed by quenching and hot press	—	—	750	0.6	142
Cl doped SnSe–SnSe_2_	Solid state reaction	—	0.42	773	0.56	143
Ag and Cl doped SnSe_2_	Bottom up	7.46	0.57	789	1.03	149
Cu embedded SnSe_2_	Solid-state reaction followed by ball mill and SPS	1.96	0.81	300	0.75	145

This material (SnSe) has the highest efficiency among the bulk materials without any doping, but it faces commercialization issues like poor mechanical stability and low thermoelectric property below ∼800 K. Besides its high *ZT*, low cost, easy fabrication, and earth-abundance, SnSe suffers the real possible application likely due to the quick oxides' formation or thermal instability.^150^ It readily forms the oxides in the range >600 °C, where it showed maximum *ZT*. Protective environment costs hamper its effectiveness as a cheap/high *ZT*. Sassi *et al.*^129^ showed thermal instability above the phase transition temperature of SnSe, and the working efficiency of the device decreased continuously over cyclic performance due to Se loss. Thus, there should be an optimized temperature range over which there is no loss of performance. Continuous research should go on in the direction to improve the thermal stability up to the temperature for which SnSe showed maximum *ZT*. Also, large phonons scattering centers exists in polycrystalline SnSe than in single crystal SnSe due to grain boundaries, yet single crystal SnSe has lower lattice thermal conductivity than the polycrystalline SnSe. Ibrahim *et al.*^151^ noted this fact and investigated the reason behind this. They systematically discarded the oxide formation and found many native defects in the single crystal.^152^ Their results reflected higher lattice thermal conductivity than the reported one.^32^ They also noted that the single crystal grown by Zhao *et al.*^32^ was not fully dense and hence may be the reason for low lattice thermal conductivity. But it still requires the study to know the exact causes of the very low lattice thermal conductivity, whether it was due to intrinsic defect or not. SnSe single crystal suffers a low mechanical strength and high growth cost as a single crystal. SnSe single crystal thermoelectric results showed inconsistencies in the different group reports' as shown in Table 2. However, the further study required to unravel the SnSe performance, where it seeks the possibilities of enhancement in *ZT*.^38^ Another phase, SnSe_2_, was also predicted by theoretical consideration as the best thermoelectric material that can achieve *ZT* around 3.5. Till date, *ZT* ∼ 1.1 is achieved in this material. Also, both materials (SnSe and SnSe_2_) showed inferior TE property in thin film form. Thus, this material demands greater attention and research to achieve predicted *ZT*. Researchers should pay attention to new approaches to improve the SnSe thermoelectric material's performance like nano-inclusion,^153^ decoupling of interrelated parameters,^119^ nano-structuring and texturing, *etc.* Simultaneously, the theoretical study should be carried out to optimize the threshold values of the parameters. Finally, tin-selenide has emerged as a futuristic material that showed the best efficiency. Both materials (SnSe and SnSe_2_) demand more research in the thin film and bulk thermoelectric form because of the inferior TE property and device stability.

### 4.3 Photodetectors (PDs)

#### 4.3.1 SnSe based PDs

**Fig. 11:**
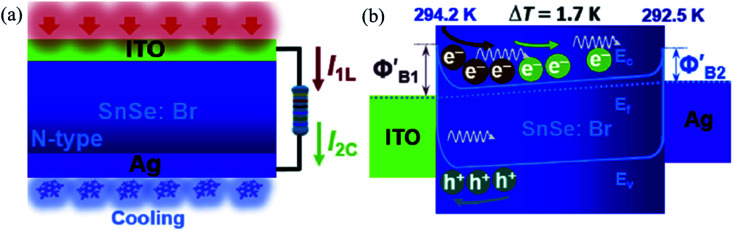
Schematic working state (a) and energy band diagram (b) of the device under the combined action of light illumination and cooling (*I*_1L_ = photovoltaic current, *I*_2C_ = thermocurrent). Reprinted with permission from. These figures has been adapted/reproduced from ref. 163 with permission from Elsevier, copyright 2019".

SnSe act as suitable optoelectronic material due to its high absorption coefficient (>10^4^ cm^−1^),^154^ low band gap and its tunable band gap over an extensive range.^155^ Solvothermal processed SnSe nanorod showed repeatable and stable photoresponse.^156^ SnSe thin film grown on the n-Si substrate showed an ultrahigh response/recovery time of 0.9/17.3 μs in a position-dependent detector.^157^ A flexible device of SnSe on the mica substrate (grown by PLD) showed high responsive behavior of the device in a longer wavelength region (370–808 nm). Also, it reflected its durability in bending test for the flexible device. The device showed the highest photoresponse of 5.5 A W^−1^ which is attributed to its shallow potential barrier with Bi_2_Te_3_ contacts (perfect band alignment) and low potential fluctuation at the interface.^158^ Under white light illumination, SnSe nanoplate and graphene composites showed very high photosensitivity (1110%) and short response time (∼1 s).^159^ The SnSe film grown on Si substrate by vapor deposition and dispersed with graphene oxide quantum dots showed improved response time and photocurrent than the SnSe film in a larger spectrum.^160^ Self-powered photodetection was demonstrated in the SnSe/Si heterostructure device, with very high detection and ultrafast response in μs range. Magnetron sputtered SnSe film on Si(100) showed a nearly ideal diode property with a reported power exponent of 0.92 and ideality factor of ∼1, which revealed a trap-free and high-performance of the device.^36^ Zhong *et al.*^161^ showed the thin film's superiority due to the Marangoni effect over other solution-based methods. The flexible and vertically fabricated Gr/SnSe/Gr device showed a responsivity of 38 mA W^−1^. Magnetron sputtered SnSe thin film on Si substrate showed broadband photoresponse in the 404–980 nm region. A maximum responsivity of 277.3 A W^−1^ was reported. This high value of responsivity was attributed to the film's trap states, which was confirmed by the nanosecond order's carrier lifetime observed in the time-resolved photoluminescence studies.^162^ Ouyang *et al.*^163^ harnessed combined photovoltaic and thermoelectrics effect to enhance the self-biased n-type SnSe:Br (ITO/n-SnSe/Ag) photodetector performance. They coupled the thermoelectric property with the material's photovoltaic property *via* maintaining the temperature difference with Peltier cooler's help at the one contact (Ag contact) of the device and another under the illumination of 760 nm radiation. The thermoelectric diffusive process accelerates photoelectrons generated under photovoltaic conditions. This is due to specific heat capacities of electrons and photonic drags under the thermal gradient resulting in enhanced performance (Fig. 11(a) and (b)). Simultaneously, in the energy band aspect, the thermoelectric effect reduced the Schottky barrier height at the ITO side and increased at the Ag side, which resulted in the de-acceleration of the photoelectrons. The combined effect enhanced the PD parameters. The light current and voltage were improved by 38.1 and 81.9%, respectively, at a cooling temperature difference of 1.5 K (the Ag side was cooler compared to the ITO side).

When the Ag side is heated w.r.t. ITO with 4.5 K difference, the opposite result was observed, *i.e.*, there was a decrease in photocurrent and voltage. Hence authors concluded that the coupling of the effect is polarity dependent. Record high value (comparable to the commercialize Ge and Si photodetectors) of responsivity (*R*) and detectivity (*D*) were reported in the nanowire SnSe.^164^ Above discussions have been summarized in Table 4.

**Table 4:** Performance characteristic of SnSe based PDs

Device structure	*λ* (nm)	Bias (V)	*R* (A W^−1^)	*D* (Jones)	Response/recovery time (s)	Ref.
ITO/SnSe/ITO	White light	5	—	—	3	165
Pd/n-Si/SnSe/Pd	780	—	—	—	0.9/17.3 × 10^−6^	157
Bi_2_Te_3_/SnSe/Bi_2_Te_3_	370	20	5.5	6 × 10^10^	—	166
Au/SnSe/graphene/Au	White light	15	—	—	1/<1	159
Ag/Si/SnSe/graphene oxide QD/Ag	650	—	—	—	0.18/0.75	160
Pd/SnSe/Si/In	850	0	0.566	4.4 × 10^10^	1.6/47.7 × 10^−6^	167
Gr/SnSe/Gr	400	1	0.038	—	∼0.18	161
Ag/Si/SnSe/Ag	404	15	277.3	7.6 × 10^11^	0.35/1.83	168
ITO/n-SnSe/Ag	760	0	3.97 × 10^−3^	—	81/122 × 10^−6^	163
Cr/Au/Si/SnSe (nanowire)/Cr/Au	830	3	1.0 × 10^4^	3.3 × 10^12^	460/520 × 10^−6^	164
Mica/In_2_Se_3_/SnSe/Au	405	5	0.350	—	0.156/0.139	169
Si/SiO_2_/WSe_2_/SnSe/Ti/Au	1064	5	6.6 × 10^−3^	—	—	170
	671		31.8 × 10^−3^		—	
	457		99 × 10^−3^		0.0082/0.0084	
PCB/Mica/SnSe (nanocrystal)/Ag	(Sunlight)	2	0.54 × 10^−3^	1.06 × 10^9^	1.5/1.7	171
PET/SnSe/Pd	404	20	1745.5	∼4.2 × 10^12^	1.7/4.7	172
	850		78.6	∼9 × 10^11^	0.23/0.27	

#### 4.3.2 SnSe_2_ based PDs

The tunable bandgap and high absorption coefficient of the SnSe_2_ have made it a potential candidate for optoelectronics applications. For the first time, Zhou *et al.*^173^ reported ultrathin SnSe_2_ flake (1.5 nm) grown by a CVD method using SnI_2_ as a precursor, which exhibited high responsitivity (*R*) 1100 A W^−1^ with meager response time in millisecond. However, the far value of power exponent (0.7), reflected its impurity and defects. Bilayered SnSe_2_ of 1T type structure with *D*_3d_ point group symmetry showed high responsivity of 0.5 A W^−1^ and a swift response time of ∼2 m s.^174^ SnSe_2_ acted as an efficient charge separator in the heterostructure devices. It collected electrons from the WSe_2_,^175^ MoS_2_ ^176^ (which have deeper conduction minima than these) so efficiently that it improved performance parameters several times better. SnSe_2_ with MoS_2_ interface resulted in enhanced responsivity from 37.3 to 9.1 × 10^3^ A W^−1^ under 500 nm illumination.^176^

**Fig. 12:**
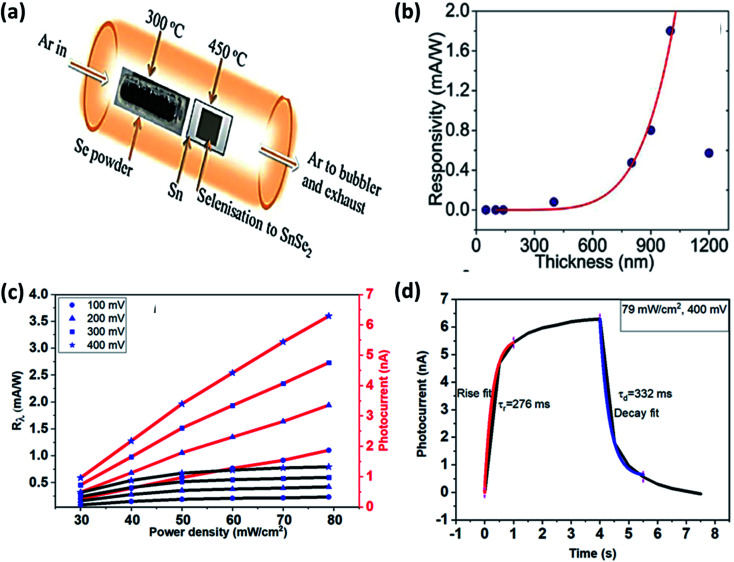
(a) Schematic of selenization of DC sputtered Sn film and (b) thickness-dependent SnSe_2_ thin films' responsivity. These figures has been adapted/reproduced from ref. 30 with permission from Nature, copyright 2017". (c) Responsivity and photocurrent with power density at different bias voltage and, (d) response and recovery time at 400 mV bias, these figures has been adapted/reproduced from ref. 65 with permission from Elsevier, copyright 2020".

Mukhokosi *et al.* studied the thickness-dependent optical properties of the SnSe_2_ thin film grown by DC sputtering followed by selenization and also studied the photodetection performance (Fig. 12(a) and (b)).^30^ Films with thickness <140 nm did not show any IR response. The film with 1200 nm thickness had a slow response (time), and responsitivity was 0.4 mA W^−1^.^30^ Mukhokosi *et al.*^177^ also reported the self-powered, organic–inorganic hybrid heterostructure PD consisting of poly (3,4-ethylene dioxythiophene):poly(styrene sulfonate) (PEDOT:PSS) and SnSe_2_. The device had responsivity in the range of 1.4–2.6 μA W^−1^. The response time improved to 1.33 s. Mukhokosi *et al.*^178^ reported highly enhanced response time and enhanced responsivity. The device was fabricated on the p-type Si substrate by DC sputtering, followed by selenization. Kumar *et al.*^65^ deposited SnSe_2_ thin-film on the soda-lime glass substrate and reported the improved responsivity at very low bias and fast response time in the IR range (Fig. 12(c) and (d)). Although the band gap was in the visible region, photodetection was observed in the IR region which was attributed to the defect/trap assisted levels lying in the forbidden region.^65^ Usually, SnSe_2_ shows high absorption coefficient^30,65^ but poor chemical stability against environmental conditions (degrade into SnSe after prolonged exposure). Therefore, Gao *et al.* used graphene (Gr) to improve the chemical stability. SnSe_2_ was sandwiched between graphene. Monolayer Gr acted as useful ohmic contacts and also had weak absorption in the UV to IR range.^179^ This sandwiched structure of SnSe_2_ with Gr showed responsivity of 1.09 × 10^3^ A W^−1^, and the response time was 30 ms. Murali *et al.* studied the substrate-induced effect on SnSe_2_ flake (i) suspended over contacts and (ii) supported on Si substrate. It showed a vast difference in rise and decay times.^180^ The suspended structure showed low gain but quicker decay than the supported one because of the device's interface-induced trap states. Field-effect transistors based on multilayer SnSe_2_ showed very high responsivity and detectivity. The device showed its potential application comparable to the other commercialized photodetector.^9^ The above discussion is summarized in Table 5.

**Table 5:** Summary of performance characteristics of SnSe_2_ based photodetectors^a^

Device structure	*λ* (nm)	Bias (V)	*R* (A W^−1^)	Detectivity (jones)	Rise/decay time (s)	Ref.
Ti/Au/Si/SnSe_2_ (flake)/Ti/Au	530	3	1100	10^10^	14.5/8.1 × 10^−3^	57
Ti/Au/Si/2LSnSe_2_/Ti/Au	633	0.1	0.5	—	2.2/3.2 × 10^−3^	174
Ni/Au/ITO/WSe_2_ (flake)/SnSe_2_ (flake)/SiO_2_/Si/Ni/Au (vertical hetero-structure)	785	0.4	1139	—	8/32 × 10^−6^	175
Cr/Au/SnSe_2_ (flake)/MoS_2_ (flake)/Cr/Au	500	1	9.1 × 10^3^	9.3 × 10^10^	0.2/0.6	181
Cr/Au/SLG/SnSe_2_ (thin film)/Cr/Au	1064	10	0.4 × 10^−3^	10^11^	2.5/3.68	30
Cr/Au/SLG/SnSe_2_ (thin film)/PEDOT:PSS/Cr/Au	1064	0	1.4–2.6 × 10^−6^	∼10^8^	1.33/1.22	177
Cr/Au/p-Si/SnSe_2_/Cr/Au	1064	10	0.16	∼1.5 × 10^9^	57/34 × 10^−6^	182
Au/SLG/SnSe_2_ (thin film)/Au	1064	0.4	0.796 × 10^−3^	5.62 × 10^7^	0.276/0.332	65
Au/Gr/SnSe_2_ (flake)/Gr/Au	532	0.5	1.09 × 10^3^	1.2 × 10^12^	30.2/27.2 × 10^−3^	183
Ni/Au/SnSe_2_ (flake)/Ni/Au (suspended structure), Ni/Au/Si/SnSe_2_ (flake)/Ni/Au (supported structure)	White light	0.1	115, 8.66 × 10^4^	∼10^13^	SED-66 s, DED-2.27 s, 53 s	180
Cr/Au/n-Si/SnSe_2_ (single crystal flake)/Cr/Au	450	3	5.11 × 10^5^	2.79 × 10^13^	—	9

a SED = single exponential decay, DED = double exponential decay.

### 4.4 Gas sensors

The gas sensor is a basic need for environment cleaning. A gas sensor is a device, which detects harmful gases and sends an alarm. There are many toxic gases in the environment (CO_2_, NO_2_, NH_3_, SO_2_, H_2_S, CH_4_, *etc.*). Gas sensing parameters are response time, recovery time, selectivity, gas concentration, temperature, detection limit, *etc.* Tin based chalcogenide (SnSe and SnSe_2_) have shown promise for gas sensing applications. A brief discussion of tin chalcogenide (SnSe and SnSe_2_) materials for gas sensing applications is described. The gas sensing mechanism of the tin chalcogenide (SnSe and SnSe_2_) gas sensor is based on the adsorption of gas molecule and charge transfer.

Physisorption-based charge transfer in tin chalcogenide (SnSe and SnSe_2_): To evaluate the interaction efficiency of the SnSe/SnSe_2_ monolayer and gas molecules, the absorption energy (*E*_a_), Hirschfeld charge transfer (*Q*), and the distance (*d*_0_) of nearest atoms between the gas molecule and the SnSe/SnSe_2_ layer were calculated. The absorbed energy is defined as:

1 *E*_a_ = *E*_total_ − *E*_gas_ − *E*_material_

*E*_gas_, *E*_material_, and *E*_total_ are the gas molecule's total energy, SnSe/SnSe_2_ monolayer, and gas molecule-SnSe/SnSe_2_ system, respectively.^184^ The adsorption properties, including adsorption energy (*E*_a_), equilibrium distance (*d*_0_), and Hirshfeld charge transfer (*Q*) for SnSe, are listed in Table 6. The adsorbed CO, CO_2_, CH_2_O, NO_2_, and SO_2_ gas molecules on the β-SnSe monolayer have the *E*_a_ values −0.202, −0.175, and −0.322, −0.829, and −0.499 eV, respectively, and the Hirshfeld charge transfer values are −0.033, −0.036, −0.085, −0.279, and −0.279 *e*, respectively.^185^

**Table 6:** The adsorption energy, equilibrium distance, and Hirshfeld charge transfer of different molecules adsorb on a β-SnSe monolayer. Reprinted with permission from^185^

Molecule	*E*_a_ (eV)	*d*_0_ (Å)	*Q* (*e*)
CO	−0.202	3.293 (C–Sn)	−0.033
CO_2_	−0.175	3.617 (C–Sn)	−0.036
CH_2_O	−0.322	3.222 (H–Sn)	−0.085
O_2_	−1.596	2.058 (O–Sn)	−0.445
NO_2_	−0.829	2.531 (O–Sn)	−0.279
SO_2_	−0.499	2.692 (O–Sn)	−0.279

The equilibrium distance of CO, CO_2_, CH_2_O, NO_2_, and SO_2_ from the β-SnSe layer is 3.293, 3.617, 3.222, 2.531, and 2.692 Å, respectively, which are greater than the C–Sn (2.15 Å), O–Sn (2.22 Å), and N–Sn (2.11 Å) bonds, indicating the process to be physisorption.^184^ However, the optimized structure shows some distortion for O_2_ on the surface of the β-SnSe sheet. The value of *E*_a_ and *Q*_c_ for O_2_ are −1.596 and −0.445 eV, respectively, which is much larger than other gas molecules adsorbed on one SnSe layer, indicating that O_2_ molecules (CO, CO_2_, CH_2_O, NO_2_, and SO_2_) are chemically absorbed in the β-SnSe layer.^185^ Adsorption of CO, CO_2_, CH_2_O on the β-SnSe layer showed lower adsorption energy and lower charge transfer values, which indicates that one β-SnSe layer is not suitable for the detection of these three molecules.^184^

**Fig. 13:**
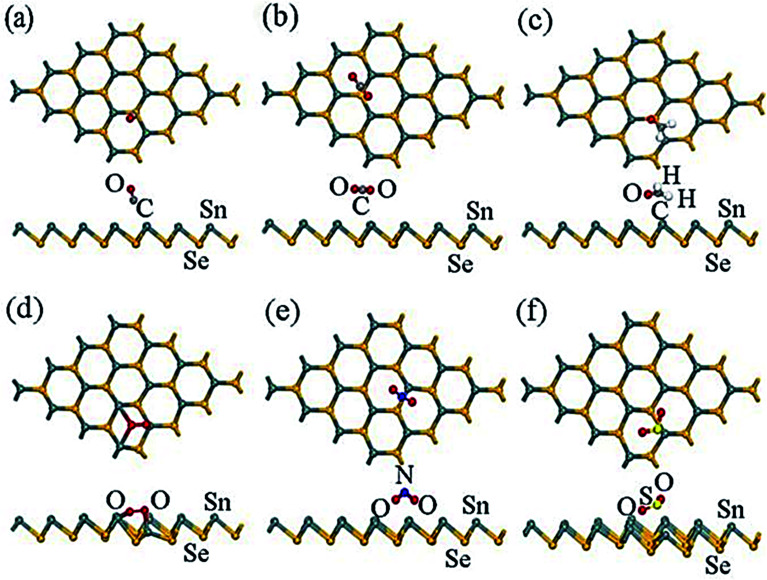
The most stable sites of optimized configurations of the adsorbate molecules: (a) CO, (b) CO_2_, (c) CH_2_O, (d) O_2_, (e) NO_2_, and (f) SO_2_ adsorbed on a β-SnSe monolayer. Most stable sites are exhibited. These figures has been adapted/reproduced^185^ with permission from MDPI, copyright 2019".

The higher absorption energy of NO_2_ (−0.829 eV) and SO_2_ (−0.499 eV) indicates that NO_2_ and SO_2_ molecules' absorption behavior for the β-SnSe layer was more potent than that of the absorption system.^185^ The charge transfer values for NO_2_ and SO_2_ are −0.279 and −0.278 eV, respectively, which show a clear charge transfer between the gas molecule and the β-SnSe layer (Fig. 13(a)–(f)).^185^ The adsorption distances for NO_2_ and SO_2_ are 2.531 Å and 2.692 Å, respectively, which is close to the Sn–O bond length range (2.22 Å to 2.66 Å).^186^ For the β-SnSe sheet, it is crucial to detect NO_2_ and SO_2_ in the gas sensor region. All the most stable energy adsorption centers are located on the Sn side of the atom of the β-SnSe sheet, which indicates that the absorbing properties of the metal atom are more substantial than that of the nonmetallic atom.^187^

#### 4.4.1 SnSe based gas sensors

**Fig. 14:**
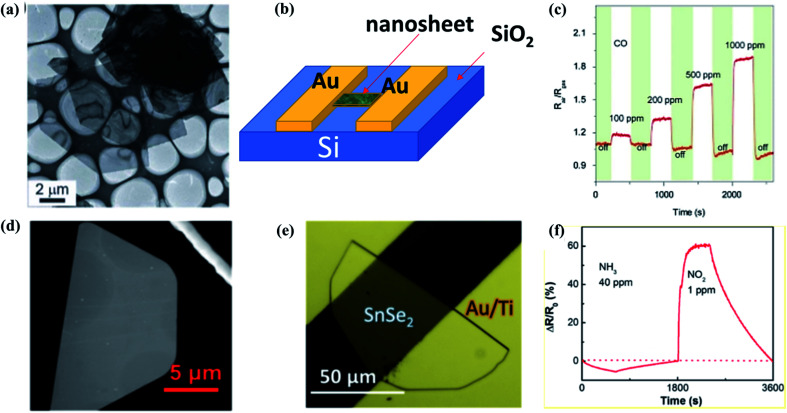
(a) Low magnification TEM images, (b) schematic structure of the device, (c) transient response of the sensor SnO_2_/SnSe to CO (100–1000 ppm) at 260 μC. These figures has been adapted/reproduced from ref. 191 with permission from Nature, copyright 2013". (d) SEM image of a semi hexagonal nanosheet of SnSe_2_, (e) optical image of a 6 nm thick SnSe_2_ gas sensor device, (f) dynamic sensing responses of the 6 nm thick SnSe_2_ resistor device measured with 405 nm laser illumination. These figures has been adapted/reproduced from ref. 61 with permission from ACS, copyright 2019".

Tin selenide (SnSe) is a member of (IVA–VIA) binary semiconductors family and has a high absorption coefficient.^188^ The bandgap of SnSe varies from 0.9 eV to 1.3 eV.^189^ SnSe has an orthorhombic crystal structure, and it has p-type conductivity with Sn-vacancies.^190^ The majority of charge carriers of SnSe are holes and have a good impact on gas sensing applications. Using Ph_3_PSe as a precursor in chemical vapor deposition, Assili *et al.*^21^ deposited orthorhombic tin selenide thin films onto three substrates. A 1% vol concentration of methane gas showed a good sensitivity at the operating temperature of about 200 °C. The sensitivity, response, and recovery times were ∼47%, ∼52 s, and ∼220 s, respectively. They observed that when SnSe was mixed with any n-type materials, it showed excellent response and recovery times at, lower operating temperature. In 2013, Wang *et al.*^191^ synthesized SnO_2_ nanoparticles decorated SnSe nanosheets *via* a facile, lost-cost, and safe solution method and had studied the gas sensing properties. For 1000 ppm CO gas, this device's response was 1.9 s at the operating temperature of 260 °C (Fig. 14(a)–(c)). Lee *et al.*^192^ prepared the SnSe_2_/SnSe heterostructure film from a thick Sn layer onto the glass substrate. The film also showed response for one ppm NO_2_ gas at room temperature, and the response value was 75%. In 2020, Wang *et al.*^193^ reported a one-step colloid method for making SnSe/SnSe_2_ heterostructures, with doping of SnSe ≈ 30, 50, and 70%, respectively. The SnSe (50%)/SnSe_2_ (50%) based sensor with an active layer thickness of 2 μm showed the highest sensitivity of 30% to 0.1 ppm NO_2_ gas at room temperature (25 °C) with a limit of detection (LOD) down to 69 ppb. The above discussion is summarized in Table 7.

**Table 7:** The comparison between the gas sensing parameter of the different structures of SnSe

Material	Structure	Gas	Gas conc. (ppm)	Temp. (°C)	Response	Response time (s)	Recovery time (s)	Ref.
SnSe	Thin film	Methane	1% vol	200	∼47%^a^	∼52	∼220	21
SnO_2_/SnSe	Nanosheet	CO	1000	260	1.9^b^	—	—	191
SnSe_2_/SnSe	Heterostructure	NO	1	RT	75%^c^	—	—	192
p-SnSe/n-SnSe_2_	Hetero-structure	NO_2_	0.1	RT	30%^b^	—	—	193

a *R* = (*R*_g_ − *R*_a_)/*R*_a_ × 100.

b *R* = *R*_a_/*R*_g_.

c *I* = (*I*_g_ − *I*_o_)/*I*_o_. *R* is called the sensor's response, *S* is called the sensor's sensitivity, *R*_g_ is the resistance of the sensor in the presence of target gas, and *R*_a_ is the sensor's resistance in the air. *I*_g_ is the current of device in presence of target gas and *I*_o_ is the current of device in presence of air.

#### 4.4.2 SnSe_2_ based gas sensors

SnSe_2_ is an n-type material due to the presence of Se-vacancies. The majority of charge carriers in SnSe_2_ are electrons. The SnSe_2_ film is an anisotropic binary material having a hexagonal structure arranged in the form of Se–Sn–Se.^194^ SnSe_2_ has a small bandgap of 1–2 eV.^30,195^ SnSe_2_ has the same electronegativity and similar structure as MoS_2_, thus it is better suited for the detection of NO_2_. 2D materials have a substantial surface to volume ratio, which significantly changes the absorption and release of gases, making them an inexpensive gas detection candidate.^196^ Given all these properties of SnSe_2_, various researchers have tried to study the properties of SnSe_2_ gas. Lee *et al.*^192^ prepared the SnSe_2_/SnSe heterostructure film on the glass substrate from a thick Sn layer. At room temperature (RT), this film showed a better response for one ppm NO_2_ gas. The response value of this film was 75%. In 2010, Popescu *et al.*^197^ prepared silver doped SnSe_2_ and Ge_2_Sb_2_Te_5_ thin films by pulsed laser deposition. Ag-Doped SnSe_2_ thin film-based gas sensor had a response of 3.26 for 500 ppm of CO gas at 500 °C. In 2018, Chen *et al.*^194^ produced SnSe_2_ nanoplate arrays and made a sensor that could detect 1% vol. of CH_4_ at the operating temperature of 200 °C. This sensor showed a response of 66.7 and response/recovery times of 78/336 s, respectively. In 2019, Moreira *et al.*^61^ investigated the NO_2_ sensing properties of CVD deposited SnSe_2_ binary layer. For one ppm of gas at the operating temperature of 50 °C, this sensor's response was 60%, and the response/recovery times of this sensor were 142/457 s (Fig. 14(d)–(f)). In 2017, Assili *et al.* demonstrated a SnSe_2_ thin film-based gas sensor. This sensor had sensitivity around 16% for 200 ppm of methane at 200 °C with the response and recovery times around 75 s and 615 s, respectively.^21^ In 2007, Popescu *et al.*^198^ reported a SnSe_2_ thin film-based gas sensor, which had a response of 300% for 8000 ppm of CH_4_ gas at 600 °C. Sanju Rani *et al.* demonstrated a SnSe/SnSe_2_ nanostructured thin film-based sensor by using of thermal evaporation technique. This sensor had response of 112% for (5 ppm) NO_2_ gas at room temperature and the response/recovery times were 15/10 s, respectively.^199^ AuPd coated SnSe_2_ thin film based NO_2_ sensor showed enhanced response of 117% for 5 ppm NO_2_ gas at room temperature. The response/recovery times of this sensor were 10/18.7 s respectively.^200^ Thus, efforts have been made to use SnSe_2_ for sensing methane as well as NO_2_. The above discussion is summarized in Table 8.

**Table 8:** The comparison between the gas sensing parameter of the different structures of SnSe_2_

Material	Structure	Gas	Gas concentration (ppm)	Temp. (°C)	Response	Response time (s)	Recovery time (s)	Ref.
SnSe_2_/SnSe	Heterostructure	NO_2_	1	RT	75%^a^	—	—	192
SnSe_2_–Ag	Thin film	CO	500	500	3.26^b^	—	—	197
SnSe_2_	Nano plate	Methane	1% vol	200	66.7^c^	78	336	194
SnSe_2_	Pristine monolayer	NO_2_	1	50	60%^b^	142	457	61
SnSe_2_	Thin-film	Methane	200	200	∼16%^c^	∼75	∼615	21
p-SnSe/n-SnSe_2_	Hetero-structure	NO_2_	0.1	RT	30%^d^	—	—	193
SnSe_2_	Thin film	CH_4_	8000	600	300%^d^	—	—	198
SnSe–SnSe_2_	Nanostructured thin film	NO_2_	5	RT	112%^e^	15	10	199
Au/Pd/SnSe_2_	Thin film	NO_2_	5	RT	117%^e^	10	18.7	200

a *S* = (*I*_g_ − *I*_o_)/*I*_o_ × 100.

b *R* = *R*_a_/*R*_g_.

c *R* = (*R*_a_ − *R*_g_)/*R*_g_ × 100.

d *S* = (*R*_g_ − *R*_a_)/*R*_a_ × 100.

e *R* = (*R*_g_/*R*_a_) × 100

### 4.5 Photocatalysis

#### 4.5.1 Photocatalytic behavior

**Fig. 15:**
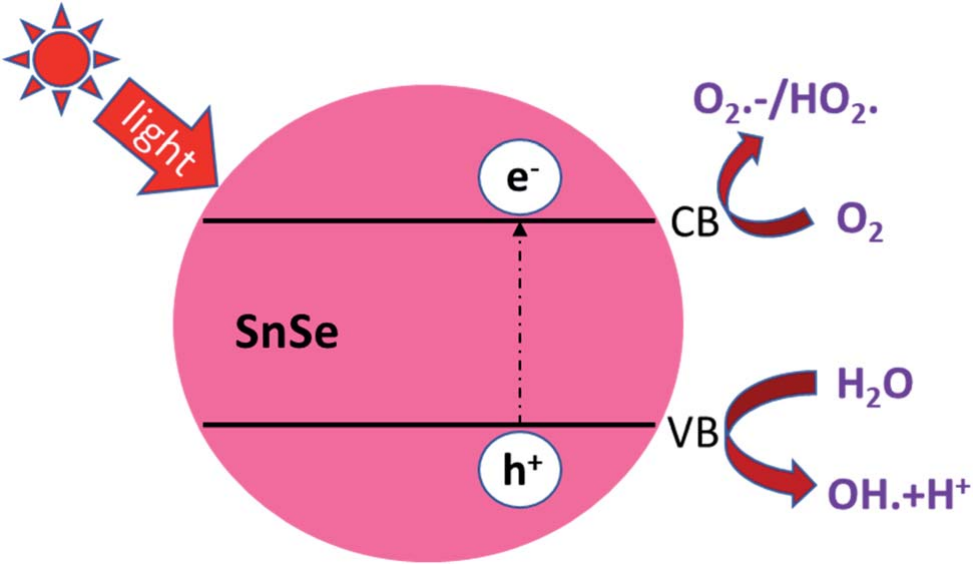
Indicative representation of photodegradation of RhB dye in the presence of UV light. In a typical experiment, the various dyes' degradation rate is checked using eqn (3). These figures has been adapted/reproduced from ref. 62 with permission from Scielo, copyright 2017".

There are several reports on the photocatalytic behavior of Sn-based materials like SnSe and SnS. The possible mechanism reported by Li Cheng and co-workers is based upon the bandgap and absorption of light. The SnS and SnSe nanofibers have a narrow bandgap of 1.01 eV and 0.90 eV, respectively. UV light excites the photoelectron and hole in the material. Now the exciting photoelectron and hole react with dissolved oxygen molecules to form oxide radicals (O_2_). Furthermore, hydroperoxyl HO_2_ and hydroxyl radical OH^−^ are formed after the protonation process (hydrogen cation). Simultaneously, the holes (h^+^) can oxidize hydroxyl radicals OH^−^ and H_2_O molecules to generate OH^−^ and hydroperoxyl HO_2_, which may break the RhB dye molecule to convert them into CO_2_ and H_2_O or in other forms as shown in Fig. 15. The dye's degradation rate is defined as 2
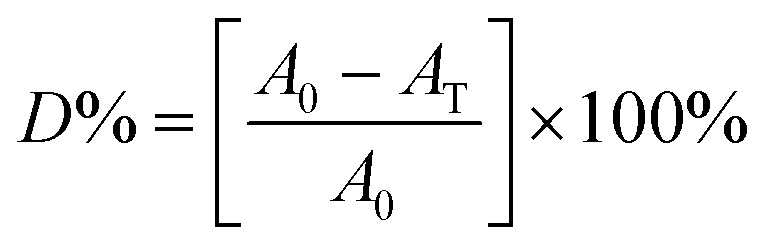


Here *A*_0_ is the absorbance of dye in the dark condition. *A*_T_ is the absorbance of dye at specific time gaps after irradiation of light of a certain wavelength. Li Cheng *et al.* used the RhB solution under UV radiation to check the SnO_2_, SnSe, and SnS nano-fibers' photocatalytic activity.^62^ The degradation curve showed a first-order kinetics equation type curve. The degradation rate for RhB was 85.90%, 92.55% and 92.86% for SnO_2_, SnS and SnSe, respectively. In another experiment, Jing group used thin films of Sn and SnSe to analyze the photodegradation of RhB.^11^ Two films SnSe_2_ and SnSe_2_/Se were made by the facile solvothermal method. The photocatalytic efficiency was measured from the formula mentioned below,3
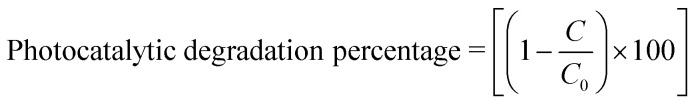
where *C* is the concentration at time *t* and *C*_0_ is the concentration at time *t*. The result showed that SnSe_2_/Se film showed better degradation, *i.e.*, around 94% compared to Sn films in 50 min. This increase in efficiency was attributed to the formation of heterojunction and the production of a larger number of electron–hole pairs and a lower recombination rate.

#### 4.5.2 SnSe composites for photocatalytic applications

Zhou Li *et al.* synthesized a composite of SnSe@SnO_2_ nanoparticles and used them in the photothermal–photocatalyst mechanism study. The mechanism of electron–hole pair generation is similar to that mentioned earlier.

The photon falls on the surface, and the elector hole pair is generated. Here, both SnSe and SnO_2_ took part in the photothermal and photocatalytic processes synchronously. In the process, the electrons from CB of SnSe travels to the CB of SnO_2_. This increases the oxidation process of photocatalysis and thus improves the photocatalytic behavior. The inner shell SnSe favors the photothermal heat efficiently.^201^ The photocatalytic activity of SnSe@SnO_2_ nanocomposite was evaluated through the degradation of methyl orange (MO) experiment under solar light's irradiation. The photocatalytic degradation of MO with SnSe, SnO_2_, SnSe@SnO_2_, and P25 (commercially named as TiO_2_ nanoparticles) was compared using the time *versus C*/*C*_0_ graph. Here, *C* is the concentration at time *t* and *C*_0_ is the concentration at time *t*. It was observed that SnSe@SnO_2_ showed a much higher degradation rate than SnSe, SnO_2_, and TiO_2_. In some other methods, blue SE-2R dye degradation was studied by making a composite.

Karamat *et al.*^202^ made SnSe composite with LaNdZr_2_O_7_ and compared the photocatalytic efficiency with SnSe and blank dye. The setup composed a 300 W Xe arc lamp (PLS-SXE300, Beijing Trusttech Co. Ltd). The degradation of blue dye was analyzed at 658 nm using a UV-Vis spectrophotometer. Before the experiment, the stability of blue dye was checked under dark and light conditions. It was confirmed that the dye was stable, and the continuous illumination does not show any loss in spectra. The photocatalytic degradation experiment was then carried out, and it was found that LaNdZr_2_O_7_/SnSe showed a better degradation efficiency. The maximum absorption intensity at wavelength 658 nm showed a decrease in absorption as the irradiation time increases. SnSe alone has demonstrated degradation efficiency of just 15.5% while mixing it with LaNdZr_2_O_7_ increased it significantly.

### 4.6 Storage devices

#### 4.6.1 Battery electrode

Tin-based alloys showed high theoretical capacity and are being considered as a very suitable material for sodium-ion batteries. Tin-based composites have a theoretical storage capacity of nearly 847 mA h g^−1^. Still, a few other composites like SnSe and SnSe_2_ showed even higher values than theoretical ones, with additional capacity contributions from conversion reactions.^14^ Considering that one molar SnSe_2_ can accomodate 7.75 molar sodium, SnSe_2_ has shown a theoretical capacity of 756 mA h g^−1^.^203^ But during the alloying process, the volume of the material expands, which is dangerous for the cycle stability; hence some composites of carbon are used to reduce this bottleneck. Tin selenide-based battery application has been divided into two categories, namely Li^−^ ion and Na^+^ ion batteries. The recent advances in SnSe_2_ for sodium-ion batteries and their results are explained in the coming section.

Zhang *et al.* synthesized SnSe_2_ based two-dimensional (2D) nanosheets using the hydrothermal technique. They achieved a theoretical capacity during the first cycle and a stable and reversible specific capacity of 515 mA h g^−1^ at 0.1 A g^−1^ after 100 cycles, which exhibited excellent performance.^204^ The SnSe and SnSe_2_ both have been used for both sodium and lithium-ion battery applications. Chen *et al.* (2018) introduced Cu in SnSe for sodium-ion battery and exhibited a capacity of 330 mA h g^−1^ at 20 A g^−1^. In a study, Xia *et al.* used electrospun SnSe with carbon nanofibers for lithium and sodium-ion battery applications and showed improved results. Kim *et al.* investigated SnSe alloy as an anode for Na-ion batteries. They exhibited excellent electrochemical performance with a high reversible capacity of 707 mA h g^−1^ and stable performance over 50 cycles.^204^ In recent time, various studies on SnSe composite has been conducted by multiple research groups to find better results due to the layered structure of SnSe and SnSe_2_ materials. Table 9 shows a summary of the above discussions.

**Table 9:** The tin selenide-based composite used for battery application and their cyclic performance

Material used	Ion transfer	Cycle rate	Ref.
Cu doped SnSe	Sodium	Retains a capacity of 330 mA h g^−1^ at 20 A g^−1^ and 304 mA h g^−1^ after 1000 cycles at 5 A g^−1^ (0.1–3.0 V *vs.* Na/Na^+^)	205
Electrospun SnSe/C nanofibers	Lithium and sodium	The discharge capacity of 405 mA h g^−1^ at 1000 mA g^−1^ after 500 cycles in lithium-ion battery and 290 mA h g^−1^ at 200 mA g^−1^ after 200 cycles in Na^+^ ion battery	60
Tin selenide/N-doped carbon composite	Lithium and sodium	For Li ion-discharge capacity 405 mA h g^−1^ after 500 cycles at a current density of 1000 mA g^−1^ for Na ion-discharge capacity of 290 mA h g^−1^ after 200 cycles at 200 mA g^−1^	206
SnSe/SnO_2_ heterostructure	Lithium	High cyclability having a capacity of 810 mA h g^−1^ after 200 cycles at a current density value of current density of 400 mA g^−1^	207
Tin diselenide hexagonal nanosheets	Lithium	Specific capacity of 795 mA h g^−1^ after 100 cycles at 100 mA g^−1^	208
SnSe_2_ nanoplate–graphene composites	Lithium	Higher storage capacity of 420 mA h g^−1^ in the first 10 cycles	55
SnSe_2_/CNTs hybrid nanostructures	Lithium	SnSe_2_/CNTs electrode showed specific capacity of 457.6 mA h g^−1^ at 0.1C and 210.3 mA h g^−1^ after 100 cycles	209
SnSe_2_/reduced graphene oxide (rGO) composite	Sodium	Exhibits an initial efficiency of 73.7%, showing a high capacity of 402.0 mA h g^−1^ after 150 cycles at 0.1 A g^−1^ with retention of 86.2%	210

Tin selenide-based composites have shown an excellent theoretical capacity. After many cycles, real practical uses of tin-based anodes in both types of batteries are still very limited. Some structural design, preparation advancement, and morphological development are needed to bring these materials to the production level.

#### 4.6.2 Supercapacitor

Supercapacitors have gained colossal consideration due to their longer operational life, higher power densities, and better safety tolerances than batteries. Zhang *et al.* synthesized tin selenide (SnSe, SnSe_2_)-based 2D nanostructures on flexible substrates for supercapacitor applications.^63^ Ni *et al.* synthesized SnSe based anode using a microwave-assisted method. They exhibited a suitable specific capacitance of 214.3 F g^−1^ at 1 A g^−1^ and rate capability of 182.8 F g^−1^ at 20 A g^−1^ with outstanding cyclic stability.^211^ Pandit *et al.* synthesized binder-free SnSe hexagonal nanosheets using a one-pot colloidal method and achieved high-performing anode material for supercapacitors. It showed a specific capacitance of 617.9 F g^−1^ at a scan rate of 2 mV s^−1^ and with fast charge–discharge cycles.^15^

### 4.7 Memory devices

The phase change from amorphous to crystalline induces a sharp change in the optical and electrical properties like reflectivity and resistivity, respectively. This change in the property is explored to store the data in the form of a bit. As this transition is reversible, this property is utilized in the rewritable data storage. Fast operating speeds, low power consumption, high energy density, large retention time, *etc.*, are the critical parameters for selecting materials for memory applications. Chalcogenides based materials play the central role in this field due to their very short time of the order of few nanoseconds of phase transition, high resistance ratio of the amorphous to the crystalline phase. Chung *et al.* investigated all phases of the tin selenide (SnSe, SnSe_2_, Sn_2_Se_3_)^7^ for memory application based on phase change and compared the result with the well-studied GeTe.^212^ SnSe_2_ is best among all phases, with high crystallization temperature (220 °C), high electrical contrast (resistance ratio of amorphous to crystalline) of 8.3 × 10^5^, *T*_g_/*T*_m_ of 0.52, which showed its potential for the memory application.^212^ Wang *et al.* demonstrated the swift recrystallization time of 20 ns for the solution-processed SnSe_2_ thin film,^213^ comparable to the commercialize Ge_2_Sb_2_Te_5_. Sun *et al.* studied the multilayer Si/SnSe_2_ film by varying the Si's thickness in steps of 4 nm for 0–20 nm, with the film's total thickness nearly constant at 100 nm.^214^ The varying thickness of the Si did not show any effect on the crystallization temperature. Still, a remarkable change in the resistance of the film with Si thickness of 12 nm {[Si (12 nm)/SnSe_2_ (5 nm)]_6_} was observed, *i.e.*, crystalline resistance increased and amorphous resistance decreased, which led to enough (more than three orders of magnitude) electrical contrast to storing information. Simultaneously increasing crystalline resistance reduced the operating current that led to lower power consumption. Multilayer with Si thickness of 12 nm (of nearly 100 nm total thickness) showed its high potential for the low power operated phase-change random access memory (PCRAM) device because of almost ten year retention time at 83 °C and low activation energy.^214^

**Fig. 16:**
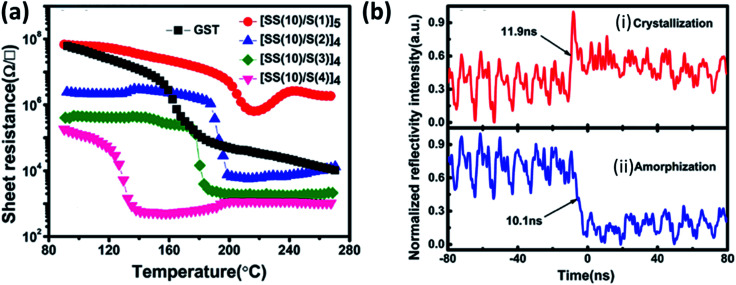
(a) The temperature dependence of resistance for the SLL SS/S and GST thin films at a constant heating rate of 10 °C min^−1^, and (b) reversible reflectivity evolution of SLL [SS(10)/S(2)] thin film induced by two consecutive picosecond laser pulses with different fluencies: (i) crystallization process and (ii) amorphization process. These figures has been adapted/reproduced from ref. 215 with permission from AIP, copyright 2016".

The difficulty in phase-change memory (PCM) application of these materials is due to high amorphous resistivity of SnSe_2_, fast crystallization speed, low thermal stability, retention of Sb rich alloy, and very low phase change temperature of Sb. Optimized design of the super lattice like (SLL) structure of SnSe_2_ and Sb ([SS(10)/S(2)]_4_) (SS stands for SnSe_2_ and S for Sb with 10 and 2 nm thickness, respectively) proved to be four times better than the commercialized Ge_2_Sb_2_Te_5_ (GST)^215^ SLL's resistance with varying temperature and phase switching times for optimized SLL is shown in Fig. 16(a) and (b)). Table 10 shows the comparison of the supremacy of SLL over GST.

**Table 10:** The supremacy of SLL over GST

Structure	Crystallization temperature (°C)	Crystallization activation energy (eV)	Year data retention at temperature (°C)	Density variation	SET/RESET operation speed (ns)
Ge_2_Sb_2_Te_5_	160	2.37	10 (75)	8.2	17.7/16.5
[SS(10)/S(2)]_4_	185	3.03	10 (116)	3.5	11.9/10.1

### 4.8 Topological insulators

Topological insulators are the materials that have insulating bulk accompanied by the protected conducting surface states. These surface states provide an impurity and defect insensitive scattering path to the electrons. The unusual metallic nature of an insulator's surface due to the changes in the insulator's topology is the signature of a topological insulator.^216^ These surface states are induced due to the high spin–orbit coupling, inverse parity of bulk band, band inversion,^217,218^ and protected by the time-reversal symmetry.^16,218^ When the crystal symmetry protects the topological insulator's surface, instead of the time-reversal symmetry protecting it, these insulators are called the topological crystalline insulator (TCI).^217^ Sun *et al.*^16^ reported that SnSe is a TCI in its native phase (without any pressure and alloying) based on the theoretical calculation using the first principle along with *ab initio* tight-binding modeling. Chen *et al.*^219^ reported the co-existence of topological states and superconducting phase at around 27 GPa of the CsCl type phase of SnSe. *Ab initio* calculation showed that this CsCl phase of SnSe has the topological states' stability against the spin–orbit coupling. SnSe provides the correlation among the superconductivity and topological electronic structures.^219^

## 5. Conclusion

This review summarizes the tin-selenide material's physical properties, phases, defects, growth mechanisms, deposition methods, and various possible applications. Tin-based compound semiconductors have attracted enormous interest in photovoltaic and optoelectronic applications due to favorable bandgap (1.0–1.3 eV) and higher absorption coefficient (10^5^ cm^−1^). The reported efficiency of tin selenide based solar cells is very low compared to other compound semiconductor materials like CIGS, CdTe, and CZTSSe. The main limiting factor for tin selenide's relative low efficiency might be defects present in absorber materials, interface trap states, and unfavorable hetero-junction interfaces. Moreover, conduction band offset (CBO) between the absorber and buffer layer plays a crucial role. It needs to be engineered in a precise manner for efficient transport of electrons toward a metal electrode. Thus, fine-tuning of elements, precise control over growth conditions, favorable band alignment between absorber and buffer can pave the way towards improving the power conversion efficiency for realizing the development of environmental-friendly low-cost next-generation photovoltaic technology. Tin selenide can be a potential futuristic material due to its versatile applications. It shows leading hands in some applications in its native crystal structure like thermoelectric. Whereas in other fields it shows competitive nature with the other materials. Tin selenide based thermoelectric materials can pave the ways to reuse waste energy. Tin selenide-based photodetector has also exhibited fast response speed with good responsivity and detectivity in the NIR range.

Moreover, flexible photodetector based on tin selenide have also gained attention due to its easy fabrication and fast device response. The device response can be improved by controlling absorber thickness and carrier collection before recombination. Another application based on tin selenide is photocathode in photoelectrochemical water splitting for green hydrogen generation. Lastly, some studies have shown that tin selenide can also be used in gas sensors, storage devices (battery electrode, supercapacitors), memory devices, topological insulators.

## Conflicts of interest

Authors declare no conflict of interest exists.

## Supplementary Material

## References

[cit1] Lokhande A. C., Qattan I. A., Lokhande C. D., Patole S. P. (2020). J. Mater. Chem. A.

[cit2] Lokhande A. C., Babar P. T., Karade V. C., Gang M. G., Lokhande V. C., Lokhande C. D., Kim J. H. (2019). J. Mater. Chem. A.

[cit3] Ali I., Suhail M., Alothman Z. A., Alwarthan A. (2018). RSC Adv..

[cit4] Wang J., Chen R., Xiang L., Komarneni S. (2018). Ceram. Int..

[cit5] Chung K. M., Wamwangi D., Woda M., Wuttig M., Bensch W. (2008). J. Appl. Phys..

[cit6] Xing G., Li Y., Fan X., Zhang L., Zheng W., Singh D. J. (2017). J. Appl. Phys..

[cit7] Bletskan D. I. (2005). J. Ovonic Res..

[cit8] Shi W., Gao M., Wei J., Gao J., Fan C., Ashalley E., Li H., Wang Z. (2018). Adv. Sci..

[cit9] Kang M., Rathi S., Lee I., Li L., Khan M. A., Lim D., Lee Y., Park J., Pham A. T., Duong A. T., Cho S., Yun S. J., Kim G.-H. (2017). J. Nanosci. Nanotechnol..

[cit10] Li Z., Guo Y., Zhao F., Nie C., Li H., Shi J., Liu X., Jiang J., Zuo S. (2020). RSC Adv..

[cit11] Li J., Zhao H., Lei Y., Yang Q., Zheng Z. (2018). Nano.

[cit12] Chung K., Wamwangi D., Woda M., Wuttig M., Bensch W. (2008). J. Appl. Phys..

[cit13] Wang X., Liu Y., Dai J., Chen Q., Huang X., Huang W. (2020). Chem.–Eur. J..

[cit14] Li Z., Ding J., Mitlin D. (2015). Acc. Chem. Res..

[cit15] Pandit B., Jadhav C. D., Chavan P. G., Tarkas H. S., Sali J. V., Gupta R. B., Sankapal B. R. (2020). IEEE Trans. Power Electron..

[cit16] Sun Y., Zhong Z., Shirakawa T., Franchini C., Li D., Li Y., Yunoki S., Chen X. Q. (2013). Phys. Rev. B: Condens. Matter Mater. Phys..

[cit17] Drozd V. E., Nikiforova I. O., Bogevolnov V. B., Yafyasov A. M., Filatova E. O., Papazoglou D. (2009). J. Phys. D: Appl. Phys..

[cit18] Zainal Z., Saravanan N., Anuar K., Hussein M. Z., Yunus W. M. M. (2004). Mater. Sci. Eng., B.

[cit19] Pallavolu M. R., Minnam Reddy V. R., Guddeti P. R., Park C. (2019). J. Mater. Sci.:
Mater. Electron..

[cit20] Narro-Rios J. S., Ramachandran M., Martínez-Escobar D., Sánchez-Juarez A. (2013). J. Semicond..

[cit21] Assili K., Gonzalez O., Alouani K., Vilanova X. (2020). Arabian J. Chem..

[cit22] Shi X., Zou J., Chen Z. (2020). Chem. Rev..

[cit23] Mukhokosi E. P., Manohar G. V. S., Nagao T., Krupanidhi S. B., Nanda K. K. (2020). Micromachines.

[cit24] Nguyen V. Q., Kim J., Cho S. (2018). J. Korean Phys. Soc..

[cit25] Chen Z., Shi X., Zhao L., Zou J. (2018). Prog. Mater. Sci..

[cit26] Shi X.-L., Chen W.-Y., Tao X., Zou J., Chen Z.-G. (2020). Mater. Horiz..

[cit27] Qin B., He W., Zhao L. D. (2020). J. Mater..

[cit28] Taniguchi M., Johnson R. L., Ghijsen J., Cardona M. (1990). Phys. Rev. B.

[cit29] Abutbul R. E., Segev E., Samuha S., Zeiri L., Ezersky V., Makov G., Golan Y. (2016). CrystEngComm.

[cit30] Mukhokosi E. P., Krupanidhi S. B., Nanda K. K. (2017). Sci. Rep..

[cit31] Chattopadhyay T., Pannetier J., Von Schnering H. G. (1986). J. Phys. Chem. Solids.

[cit32] Zhao L. D., Lo S. H., Zhang Y., Sun H., Tan G., Uher C., Wolverton C., Dravid V. P., Kanatzidis M. G. (2014). Nature.

[cit33] Ding Y., Xiao B., Tang G., Hong J. (2017). J. Phys. Chem. C.

[cit34] Zhou M., Chen X., Li M., Du A. (2017). J. Mater. Chem. C.

[cit35] Gonzalez J. M., Oleynik I. I. (2016). Phys. Rev. B.

[cit36] Hao L., Wang Z., Xu H., Yan K., Dong S., Liu H., Du Y., Wu Y., Liu Y., Dong M. (2019). 2D Mater..

[cit37] Xing G., Li Y., Fan X., Zhang L., Zheng W., Singh D. J. (2017). J. Appl. Phys..

[cit38] Chunhong L., Donglin G., Li K., Saho B., Chen D., Ma Y., Sun J. (2018). Phys. B Phys. Condens. Matter.

[cit39] Shi X., Tao X., Zou J., Chen Z. (2020). Adv. Sci..

[cit40] Huang Y., Wang C., Chen X., Zhou D., Du J., Wang S., Ning L. (2017). RSC Adv..

[cit41] Duvjir G., Min T., Thi Ly T., Kim T., Duong A. T., Cho S., Rhim S. H., Lee J., Kim J. (2017). Appl. Phys. Lett..

[cit42] Nariya B. B., Dasadia A. K., Bhayani M. K., Patel A. J., Jani A. R. (2009). Chalcogenide Lett..

[cit43] Shanmugam G., Deshpande U. P., Sharma A., Shirage P. M., Bhobe P. A. (2018). J. Phys. Chem. C.

[cit44] Zhou Y., Li W., Wu M., Zhao L. D., He J., Wei S. H., Huang L. (2018). Phys. Rev. B.

[cit45] Song L., Zhang J., Iversen B. B. (2019). J. Mater. Chem. A.

[cit46] Indirajith R., Srinivasan T. P., Ramamurthi K., Gopalakrishnan R. (2010). Curr. Appl. Phys..

[cit47] Gong Y., Chang C., Wei W., Liu J., Xiong W., Chai S., Li D., Zhang J., Tang G. (2018). Scr. Mater..

[cit48] Martínez-Escobar D., Ramachandran M., Sánchez-Juárez A., Narro Rios J. S. (2013). Thin Solid Films.

[cit49] Davitt F., Manning H. G., Robinson F., Hawken S. L., Biswas S., Petkov N., van Druenen M., Boland J. J., Reid G., Holmes J. D. (2020). Adv. Mater. Interfaces.

[cit50] Bhatt V. P., Gireesan K., Pandya G. R. (1989). J. Cryst. Growth.

[cit51] Duong A. T., Nguyen V. Q., Duvjir G., Duong V. T., Kwon S., Song J. Y., Lee J. K., Lee J. E., Park S., Min T., Lee J., Kim J., Cho S. (2016). Nat. Commun..

[cit52] Li J., Liu W., Chen C., Zhao X., Qiu Z., Xu H., Sheng F., Hu Q., Zheng Y., Lin M., Pennycook S. J., Su C., Lu J. (2019). J. Mater. Chem. A.

[cit53] Jiang J., Wong C. P. Y., Zou J., Li S., Wang Q., Chen J., Qi D., Wang H., Eda G., Chua D. H. C., Shi Y., Zhang W., Wee A. T. S. (2017). 2D Mater..

[cit54] Wang Z., Li F., Guo J., Ma C., Song Y., He Z., Liu J., Zhang Y., Li D., Zhang H. (2020). Adv. Opt. Mater..

[cit55] Choi J., Jin J., Jung I. G., Kim J. M., Kim H. J., Son S. U. (2011). Chem. Commun..

[cit56] Li X., Li L., Zhao H., Ruan S., Zhang W., Yan P., Sun Z., Liang H., Tao K. (2019). Nanomaterials.

[cit57] Zhou X., Gan L., Tian W., Zhang Q., Jin S., Li H., Bando Y., Golberg D., Zhai T. (2015). Adv. Mater..

[cit58] Ma D. W., Cheng C. (2013). J. Nanosci. Nanotechnol..

[cit59] Franzman M. A., Schlenker C. W., Thompson M. E., Brutchey R. L. (2010). J. Am. Chem. Soc..

[cit60] Xia J., Yuan Y., Yan H., Liu J., Zhang Y., Liu L., Zhang S., Li W., Yang X., Shu H., Wang X., Cao G. (2020). J. Power Sources.

[cit61] Moreira Ó. L. C., Cheng W. Y., Fuh H. R., Chien W. C., Yan W., Fei H., Xu H., Zhang D., Chen Y., Zhao Y., Lv Y., Wu G., Lv C., Arora S. K., Ó Coileáin C., Heng C., Chang C. R., Wu H. C. (2019). ACS Sens..

[cit62] Cheng L., Li D., Dong X., Ma Q., Yu W., Wang X., Yu H., Wang J., Liu G., Cheng L., Li D., Dong X., Ma Q., Yu W., Wang X., Yu H., Wang J., Liu G. (2017). Mater. Res..

[cit63] Zhang C., Yin H., Han M., Dai Z., Pang H., Zheng Y., Lan Y. Q., Bao J., Zhu J. (2014). ACS Nano.

[cit64] Feng X., Hu Y., Zhai J., Wang C., Song S., Song Z. (2014). J. Appl. Phys..

[cit65] Kumar M., Rani S., Pandey A., Gour K. S., Husale S., Singh P., Singh V. N. (2020). J. Alloys Compd..

[cit66] Powalla M., Paetel S., Ahlswede E., Wuerz R., Wessendorf C. D., Magorian Friedlmeier T. (2018). Appl. Phys. Rev..

[cit67] Cui X., Sun K., Huang J., Yun J. S., Lee C. Y., Yan C., Sun H., Zhang Y., Xue C., Eder K., Yang L., Cairney J. M., Seidel J., Ekins-Daukes N. J., Green M., Hoex B., Hao X. (2019). Energy Environ. Sci..

[cit68] Li X., Zhuang D., Zhang N., Zhao M., Yu X., Liu P., Wei Y., Ren G. (2019). J. Mater. Chem. A.

[cit69] Son D. H., Kim S. H., Kim S. Y., Kim Y. I., Sim J. H., Park S. N., Jeon D. H., Hwang D. K., Sung S. J., Kang J. K., Yang K. J., Kim D. H. (2019). J. Mater. Chem. A.

[cit70] Singh O. P., Gour K. S., Parmar R., Singh V. N. (2018). J. Nanosci. Nanotechnol..

[cit71] Pejjai B., Minnam Reddy V. R., Seku K., Pallavolu M. R., Park C. (2018). New J. Chem..

[cit72] Rühle S. (2016). Sol. Energy.

[cit73] Shinde D. V., Min S. K., Sung M. M., Shrestha N. K., Mane R. S., Han S. H. (2014). Mater. Lett..

[cit74] Mathews N. R. (2012). Sol. Energy.

[cit75] Singh J. P., Bedi R. K. (1990). Jpn. J. Appl. Phys..

[cit76] Delice S., Isik M., Gullu H. H., Terlemezoglu M., Bayrakli Surucu O., Parlak M., Gasanly N. M. (2019). J. Phys. Chem. Solids.

[cit77] Minnam Reddy V. R., Lindwall G., Pejjai B., Gedi S., Kotte T. R. R., Sugiyama M., Liu Z. K., Park C. (2018). Sol. Energy Mater. Sol. Cells.

[cit78] Nakamura M., Yamaguchi K., Kimoto Y., Yasaki Y., Kato T., Sugimoto H. (2019). IEEE J. Photovoltaics.

[cit79] Park J., Yoo H., Karade V., Gour K. S., Choi E., Kim M., Hao X., Shin S. J., Kim J., Shim H., Kim D., Kim J. H., Yun J., hyeok Kim J. (2020). J. Mater. Chem. A.

[cit80] Gour K. S., Parmar R., Kumar R., Singh V. N. (2019). J. Nanosci. Nanotechnol..

[cit81] Dhankhar M., Pal Singh O., Singh V. N. (2014). Renewable Sustainable Energy Rev..

[cit82] Fernandes P. A., Sousa M. G., Salomé P. M. P., Leitão J. P., Da Cunha A. F. (2013). CrystEngComm.

[cit83] Abd El-Rahman K. F., Darwish A. A. A., El-Shazly E. A. A. (2014). Mater. Sci. Semicond. Process..

[cit84] Makori N. E., Amatalo I. A., Karimi P. M., Njoroge W. K. (2015). Int. J. Energy Eng..

[cit85] Wang W., Winkler M. T., Gunawan O., Gokmen T., Todorov T. K., Zhu Y., Mitzi D. B. (2014). Adv. Energy Mater..

[cit86] Jin Q., Jiang S., Zhao Y., Wang D., Qiu J., Tang D. M., Tan J., Sun D. M., Hou P. X., Chen X. Q., Tai K., Gao N., Liu C., Cheng H. M., Jiang X. (2019). Nat. Mater..

[cit87] Hu L., Zhang Y., Wu H., Liu Y., Li J., He J., Ao W., Liu F., Pennycook S. J., Zeng X. (2018). Adv. Funct.
Mater..

[cit88] Markov M., Hu X., Liu H. C., Liu N., Poon S. J., Esfarjani K., Zebarjadi M. (2018). Sci. Rep..

[cit89] Nagai H., Hamada H., Hayashi K., Miyazaki Y. (2019). J. Electron. Mater..

[cit90] Yang J., Zhang G., Yang G., Wang C., Wang Y. X. (2015). J. Alloys Compd..

[cit91] Shi G., Kioupakis E. (2015). J. Appl. Phys..

[cit92] Kutorasinski K., Wiendlocha B., Kaprzyk S., Tobola J. (2015). Phys. Rev. B: Condens. Matter Mater. Phys..

[cit93] Zhao L., Tan G., Hao S., He J., Pei Y., Chi H., Wang H., Gong S., Xu H., Dravid V. P., Uher C., Snyder G. J., Wolverton C., Kanatzidis M. G. (2016). Science.

[cit94] Wei P. C., Bhattacharya S., Liu Y. F., Liu F., He J., Tung Y. H., Yang C. C., Hsing C. R., Nguyen D. L., Wei C. M., Chou M. Y., Lai Y. C., Hung T. L., Guan S. Y., Chang C. S., Wu H. J., Lee C. H., Li W. H., Hermann R. P., Chen Y. Y., Rao A. M. (2019). ACS Omega.

[cit95] Jin M., Tang Z., Jiang J., Zhang R., Zhou L., Zhao S., Chen Y., Chen Y., Wang X., Li R. (2020). Mater. Res. Bull..

[cit96] Chang C., Wu M., He D., Pei Y., Wu C. F., Wu X., Yu H., Zhu F., Wang K., Chen Y., Huang L., Li J. F., He J., Zhao L. D. (2018). Science.

[cit97] Patel S., Chaki S. H., Vinodkumar P. C. (2019). Mater. Res. Express.

[cit98] Jayaraman A., Molli M., Kamisetti V. (2015). AIP Conf. Proc..

[cit99] Tang Y., Shen L., Chen Z., Sun L., Liu W., Liu J., Deng S. (2019). Phys. B Phys. Condens. Matter.

[cit100] Jin M., Shao H., Hu H., Li D., Xu J., Liu G., Shen H., Xu J., Jiang H., Jiang J. (2017). J. Cryst. Growth.

[cit101] Peng K., Wu H., Yan Y., Guo L., Wang G., Lu X., Zhou X. (2017). J. Mater. Chem. A.

[cit102] Lee Y. K., Luo Z., Cho S. P., Mercouri G., Lee Y. K., Luo Z., Cho S. P., Kanatzidis M. G., Chung I. (2019). Joule.

[cit103] Zhang M., Wang D., Chang C., Lin T., Wang K., Zhao L. D. (2019). J. Mater. Chem. C.

[cit104] Sassi S., Candolfi C., Vaney J., Ohorodniichuk V., Masschelein P., Dauscher A., Lenoir B., Sassi S., Candolfi C., Vaney J., Ohorodniichuk V., Masschelein P., Dauscher A. (2014). Appl. Phys. Lett..

[cit105] Kumar M., Rani S., Singh Y., Singh V. N. (2019). J. Nanosci. Nanotechnol..

[cit106] Shi X., Zheng K., Hong M., Liu W., Moshwan R., Wang Y., Qu X., Chen Z. G., Zou J. (2018). Chem. Sci..

[cit107] Zhao L. D., Chang C., Tan G., Kanatzidis M. G. (2016). Energy Environ. Sci..

[cit108] Gainza J., Alonso A., Nemes N. M., Gainza J., Serrano-sa F., Simon F., Martı L., Alonso A., Nemes N. M. (2020). Cell reports Phys. Sci..

[cit109] Cho J. Y., Siyar M., Bae S. H., Mun J. S., Kim M. Y., Hong S. H., Park C. (2020). Bull. Mater. Sci..

[cit110] Chere E. K., Zhang Q., Dahal K., Cao F., Mao J., Ren Z. (2016). J. Mater. Chem. A.

[cit111] Chen Y. X., Ge Z. H., Yin M., Feng D., Huang X. Q., Zhao W., He J. (2016). Adv. Funct. Mater..

[cit112] Shi X., Wu A., Feng T., Zheng K., Liu W., Sun Q., Hong M., Pantelides S. T., Chen Z. G., Zou J. (2019). Adv. Energy Mater..

[cit113] Chien C. H., Chang C. C., Chen C. L., Tseng C. M., Wu Y. R., Wu M. K., Lee C. H., Chen Y. Y. (2017). RSC Adv..

[cit114] Luo Y., Cai S., Hua X., Chen H., Liang Q., Du C., Zheng Y., Shen J., Xu J., Wolverton C., Dravid V. P., Yan Q., Kanatzidis M. G. (2019). Adv. Energy Mater..

[cit115] Wang S., Su X., Bailey T. P., Hu T., Zhang Z., Tan G., Yan Y., Liu W., Uher C., Tang X. (2019). RSC Adv..

[cit116] Zhang X., Wang Y., Zhang G., Wang C., Yan Y. (2019). J. Alloys Compd..

[cit117] Chu F., Zhang Q., Zhou Z., Hou D., Wang L., Jiang W. (2018). J. Alloys Compd..

[cit118] Lu W., Li S., Xu R., Zhang J., Li D., Feng Z., Zhang Y., Tang G. (2019). ACS Appl. Mater. Interfaces.

[cit119] Yang G., Sang L., Li M., Kazi Nazrul Islam S. M., Yue Z., Liu L., Li J., Mitchell D. R. G., Ye N., Wang X. (2020). ACS Appl. Mater. Interfaces.

[cit120] Zhang Q. K., Ning S. T., Qi N., Chen Z. Q., Tang X. F., Chen Z. Y. (2019). J. Appl. Phys..

[cit121] Chandra S., Biswas K. (2019). J. Am. Chem. Soc..

[cit122] Urmila K. S., Namitha T. A., Rajani J., Philip R. R., Pradeep B. (2016). J. Semicond..

[cit123] Nair P. K., Martínez A. K., Angelmo A. R. G., Salgado E. B., Nair M. T. S. (2018). Semicond. Sci. Technol..

[cit124] Suen C. H., Shi D., Su Y., Zhang Z., Chan C. H., Tang X., Li Y., Lam K. H., Chen X., Huang B. L., Zhou X. Y., Dai J. Y. (2017). J. Mater..

[cit125] Burton M. R., Liu T., Mcgettrick J., Mehraban S., Baker J., Pockett A., Watson T., Fenwick O., Carnie M. J. (2018). Adv. Mater..

[cit126] Song L., Zhang J., Iversen B. B. (2019). J. Mater. Chem. A.

[cit127] Yan Z., Zhao Y., Liu D., Zhang Y., Xue C., Zhang Z., Zheng Y., Cui J. (2020). RSC Adv..

[cit128] Wei P., Bhattacharya S., Liu Y.-F., Liu F., He J., Tung Y., Yang C.-C., Hsing C.-R., Nguyen D.-L., Wei C.-M., Chou M.-Y., Lai Y.-C., Hung T.-L., Guan S.-Y., Chang C.-S., Wu H.-J., Lee C.-H., Li W.-H., Hermann R. P., Chen Y.-Y., Rao A. M. (2019). ACS Omega.

[cit129] Sassi S., Candolfi C., Vaney J.-B., Ohorodniichuk V., Masschelein P., Dauscher A., Lenoir B. (2014). Appl. Phys. Lett..

[cit130] Llorca J., Cadavid D., Iba M. (2020). ACS Appl. Mater. Interfaces.

[cit131] Chandra S., Dutta P., Biswas K. (2020). ACS Appl. Energy Mater..

[cit132] Cai B., Zhuang H. L., Tang H., Li J. F. (2019). Nano Energy.

[cit133] Cha J., Zhou C., Lee Y. K., Cho S. P., Chung I. (2019). ACS Appl. Mater. Interfaces.

[cit134] Lee Y. K., Luo Z., Cho S. P., Kanatzidis M. G., Chung I. (2019). Joule.

[cit135] Ho C., Lin W., Chao L., Lee K., Inagaki J., Hsueh H. (2020). ACS Appl. Energy Mater..

[cit136] Zhong Y., Zhang L., Linseis V., Qin B., Chen W., Zhao L., Zhu H. (2020). Nano Energy.

[cit137] Sun B. Z., Ma Z., He C., Wu K. (2015). Phys. Chem. Chem. Phys..

[cit138] Saha S., Banik A., Biswas K. (2016). Chem.–Eur. J..

[cit139] Xu P., Fu T., Xin J., Liu Y., Ying P., Zhao X., Pan H., Zhu T. (2017). Sci. Bull..

[cit140] Luo Y., Zheng Y., Luo Z., Hao S., Du C., Liang Q., Li Z., Khor K. A., Hippalgaonkar K., Xu J., Yan Q., Wolverton C., Kanatzidis M. G. (2018). Adv. Energy Mater..

[cit141] Li F., Zheng Z., Li Y., Wang W., Li J. F., Li B., Zhong A., Luo J., Fan P. (2017). J. Mater. Sci..

[cit142] Wu Y., Li W., Faghaninia A., Chen Z., Li J., Zhang X., Gao B., Lin S., Zhou B., Jain A., Pei Y. (2017). Mater. Today Phys..

[cit143] Shu Y., Su X., Xie H., Zheng G., Liu W., Yan Y., Luo T., Yang X., Yang D., Uher C., Tang X. (2018). ACS Appl. Mater. Interfaces.

[cit144] Liu C., Huang Z., Wang D., Wang X., Miao L., Wang X., Wu S., Toyama N., Asaka T., Chen J., Nishibori E., Zhao L.-D. (2019). J. Mater. Chem. A.

[cit145] Wang J., Jia X., Lou S., Li G., Zhou S. (2020). ACS Omega.

[cit146] Yin D., Liu Y., Dun C., Carroll D. L., Swihart M. T. (2018). Nanoscale.

[cit147] Chen J., Hamann D. M., Choi D., Poudel N., Shen L., Shi L., Johnson D. C., Cronin S. (2018). Nano Lett..

[cit148] Duong A. T., Nguyen D. L., Nguyen M. N., Nguyen T. M. H., Nguyen A. D., Pham A. T., Ullah F., Tahir Z., Kim Y. S., Trung D. Q., Nguyen T., Van Bui H., Das R., Huy P. T., Cho S. (2019). Mater. Res. Express.

[cit149] Zhang X., Liu D., Yang L., Zhou L., You T. (2015). J. Mater. Chem. A.

[cit150] Li Y., He B., Heremans J. P., Zhao J., He B., Heremans J. P. (2016). J. Alloys Compd..

[cit151] Ibrahim D., Vaney J., Sassi S., Candolfi C., Ohorodniichuk V., Levinsky P., Semprimoschnig C., Dauscher A., Lenoir B. (2017). Appl. Phys. Lett..

[cit152] Wei P. C., Bhattacharya S., He J., Neeleshwar S., Podila R., Chen Y. Y., Rao A. M. (2016). Nature.

[cit153] Gupta R., Bera C. (2020). Nano Express.

[cit154] Makori N. E., Amatalo I. A., Karimi P. M., Njoroge W. K. (2014). Am. J. Condens. Matter Phys..

[cit155] Ghobadi N., Gholami Hatam E. (2019). Opt. Quantum Electron..

[cit156] Pawbake A. S., Jadkar S. R., Late D. J. (2016). Mater. Res. Express.

[cit157] Hao L., Xu H., Dong S., Du Y., Luo L., Zhang C., Liu H., Wu Y., Liu Y. (2018). IEEE Electron Device Lett..

[cit158] Yao J., Zheng Z., Yang G. (2017). Adv. Funct. Mater..

[cit159] Liu J., Huang Q., Zhang K., Xu Y., Guo M., Qian Y., Huang Z., Lai F., Lin L. (2017). Nanoscale Res. Lett..

[cit160] Yao H., Luo S., Duesberg G. S., Qi X., Lu D., Yue C., Zhong J. (2018). AIP Adv..

[cit161] Zhong Y., Zhang L., Sun M., Wang M., Chen W., Lin S., Xie D., Zhu H. (2019). Mater. Today Energy.

[cit162] Hao L., Du Y., Wang Z., Wu Y., Xu H., Dong S., Liu H., Liu Y., Xue Q., Han Z., Yan K., Dong M. (2020). Nanoscale.

[cit163] Ouyang B., Chang C., Zhao L., Lin Z., Yang Y. (2019). Nano Energy.

[cit164] Zheng D., Fang H., Long M., Wu F., Wang P., Gong F., Wu X., Ho J. C., Liao L., Hu W. (2018). ACS Nano.

[cit165] Pawbake A. S., Jadkar S. R., Late D. J. (2016). Mater. Res. Express.

[cit166] Yao J., Zheng Z., Yang G. (2017). Adv. Funct. Mater..

[cit167] Hao L., Wang Z., Xu H., Yan K., Dong S., Liu H., Du Y., Wu Y., Liu Y., Dong M. (2019). 2D Mater.

[cit168] Hao L., Du Y., Wang Z., Wu Y., Xu H., Dong S., Liu H., Liu Y., Xue Q., Han Z., Yan K., Dong M. (2020). Nanoscale.

[cit169] Li X.-Z., Wang Y.-F., Xia J., Meng X.-M. (2019). Nanoscale Adv..

[cit170] Jia Z., Xiang J., Wen F., Yang R., Hao C., Liu Z. (2016). ACS Appl. Mater. Interfaces.

[cit171] Patel K., Chauhan P., Patel A. B., Solanki G. K., Patel K. D., Pathak V. M. (2020). ACS Appl. Nano Mater..

[cit172] Xu H., Hao L., Liu H., Dong S., Wu Y., Liu Y., Cao B., Wang Z., Ling C., Li S., Xu Z., Xue Q., Yan K. (2020). ACS Appl. Mater. Interfaces.

[cit173] Zhou X., Gan L., Tian W., Zhang Q., Jin S., Li H., Bando Y., Golberg D., Zhai T. (2015). Adv. Mater..

[cit174] Yu P., Yu X., Lu W., Lin H., Sun L., Du K., Liu F., Fu W., Zeng Q., Shen Z., Jin C., Wang Q. J., Liu Z. (2016). Adv. Funct. Mater..

[cit175] Murali K., Majumdar K. (2018). IEEE Trans. Electron Devices.

[cit176] Zhou X., Zhou N., Li C., Song H., Zhang Q., Hu X., Gan L., Li H., Lü J., Luo J., Xiong J., Zhai T. (2017). 2D Mater.

[cit177] Mukhokosi E. P., Krupanidhi S. B., Nanda K. K. (2018). Phys. Status Solidi A.

[cit178] Mukhokosi E. P., Roul B., Krupanidhi S. B., Nanda K. K. (2019). ACS Appl. Mater. Interfaces.

[cit179] Gao W., Zheng Z., Li Y., Zhao Y., Xu L., Deng H., Li J. (2019). Nanoscale.

[cit180] Krishna M., Kallatt S., Majumdar K. (2018). Nanotechnology.

[cit181] Zhou X., Zhou N., Li C., Song H., Zhang Q., Hu X., Gan L., Li H., Lü J., Luo J., Xiong J., Zhai T. (2017). 2D Mater..

[cit182] Mukhokosi E. P., Roul B., Krupanidhi S. B., Nanda K. K. (2019). ACS Appl. Mater. Interfaces.

[cit183] Gao W., Zheng Z., Li Y., Zhao Y., Xu L., Deng H., Li J. (2019). Nanoscale.

[cit184] Wang J., Yang G. F., Xue J. J., Lei J. M., Chen D. J., Lu H., Zhang R., Zheng Y. D. (2018). IEEE Electron Device Lett..

[cit185] Liu T., Qin H., Yang D., Zhang G. (2019). Coatings.

[cit186] Brown I. D. (1974). J. Solid State Chem..

[cit187] Feng C., Qin H., Yang D., Zhang G. (2019). Materials.

[cit188] He X., Shen H., Wang W., Wang Z., Zhang B., Li X. (2013). J. Alloys Compd..

[cit189] Shi G., Kioupakis E. (2015). Nano Lett..

[cit190] Lefebvre I., Szymanski M. A., Olivier-Fourcade J., Jumas J. C. (1998). Phys. Rev. B.

[cit191] Wang J.-J., Lv A.-F., Wang Y.-Q., Cui B., Yan H.-J., Hu J.-S., Hu W.-P., Guo Y.-G., Wan L.-J. (2013). Sci. Rep..

[cit192] Lee L., Chen C. W., Manikandan A., Lee S. H., Wang Z. M., Chueh Y. L. (2018). Nano Energy.

[cit193] Wang X., Liu Y., Dai J., Chen Q., Huang X., Huang W. (2020). Chem.–Eur. J..

[cit194] Chen M., Li Z., Li W., Shan C., Li W., Li K., Gu G., Feng Y., Zhong G., Wei L., Yang C. (2018). Nanotechnology.

[cit195] Subramanian B., Mahalingam T., Sanjeeviraja C., Jayachandran M., Chockalingam M. J. (1999). Thin Solid Films.

[cit196] Choi S. J., Kim I. D. (2018). Recent Developments in 2D Nanomaterials for Chemiresistive-Type Gas Sensors Electronic Mater. Lett..

[cit197] Popescu M., Velea A., Sava F., Lőrinczi A., Tomescu A., Simion C., Matei E., Socol G., Mihailescu I. N., Andonie A., Stamatin I. (2010). Phys. Status Solidi.

[cit198] Popescu M., Sava F., Lorinczi A., Socol G., Mihǎilescu I. N., Tomescu A., Simion C. (2007). J. Non-Cryst. Solids.

[cit199] Rani S., Kumar M., Singh Y., Tomar M., Sharma A., Gupta V., Singh V. N. (2021). J. Nanosci. Nanotechnol..

[cit200] Rani S., Kumar M., Singh Y., Singh V. N. (2021). J. Nanosci. Nanotechnol..

[cit201] Li Z., Sun L., Liu Y., Yu D., Wang Y., Sun Y., Yu M. (2019). Environ. Sci. Nano.

[cit202] Karamat M., Fahad Ehsan M., Naeem Ashiq M., Ijaz S., Najam-ul-Haq M., Hamid S., Bahnemann H.N. (2019). Appl. Surf. Sci..

[cit203] Zhang F., Xia C., Zhu J., Ahmed B., Liang H., Velusamy D. B., Schwingenschlögl U., Alshareef H. N. (2016). Adv. Energy Mater..

[cit204] Kim Y., Kim Y., Park Y., Jo Y. N., Kim Y. J., Choi N. S., Lee K. T. (2015). Chem. Commun..

[cit205] Chen R., Li S., Liu J., Li Y., Ma F., Liang J., Chen X., Miao Z., Han J., Wang T., Li Q. (2018). Electrochim. Acta.

[cit206] Shaji N., Santhoshkumar P., Kang H. S., Nanthagopal M., Park J. W., Praveen S., Sim G. S., Senthil C., Lee C. W. (2020). J. Alloys Compd..

[cit207] Chen K., Wang X., Wang G., Wang B., Liu X., Bai J., Wang H. (2018). Chem. Eng. J..

[cit208] Yu Q., Wang B., Wang J., Hu S., Hu J., Li Y. (2020). Front. Chem..

[cit209] Chen H., Jia B.-E., Lu X., Guo Y., Hu R., Khatoon R., Jiao L., Leng J., Zhang L., Lu J. (2019). Chem.–Eur. J..

[cit210] Wang T., Yang K., Shi J., Zhou S., Mi L., Li H., Chen W. (2020). J. Energy Chem..

[cit211] Ni D., Chen Y., Yang X., Liu C., Cai K. (2018). J. Alloys Compd..

[cit212] Chung K. M., Wamwangi D., Woda M., Wuttig M., Bensch W. (2008). J. Appl. Phys..

[cit213] Wang R. Y., Caldwell M. A., Gnana R., Jeyasingh D., Aloni S., Shelby R. M., Wang R. Y., Caldwell M. A., Gnana R., Jeyasingh D., Aloni S., Shelby R. M., Wong H. P., Milliron D. J. (2011). J. Appl. Phys..

[cit214] Journal A. I., Sun M., Hu Y., Shen B., Zhai J., Song S. (2012). Integr. Ferroelectr..

[cit215] Wu W., He Z., Chen S., Zhai J., Liu X., Lai T., Song S., Song Z. (2016). J. Appl. Phys..

[cit216] Moore J. E. (2010). Nature.

[cit217] Shen J., Cha J. J. (2014). Nanoscale.

[cit218] Müchler L., Zhang H., Chadov S., Yan B., Casper F., Kübler J., Zhang S. C., Felser C. (2012). Angew. Chem., Int. Ed..

[cit219] Chen X., Lu P., Wang X., Zhou Y., An C., Zhou Y., Xian C., Gao H., Guo Z., Park C., Hou B., Peng K., Zhou X., Sun J., Xiong Y., Yang Z., Xing D., Zhang Y. (2017). Phys. Rev. B.

